# The KdmB-EcoA-RpdA-SntB chromatin complex binds regulatory genes and coordinates fungal development with mycotoxin synthesis

**DOI:** 10.1093/nar/gkac744

**Published:** 2022-09-12

**Authors:** Betim Karahoda, Lakhansing Pardeshi, Mevlut Ulas, Zhiqiang Dong, Niranjan Shirgaonkar, Shuhui Guo, Fang Wang, Kaeling Tan, Özlem Sarikaya-Bayram, Ingo Bauer, Paul Dowling, Alastair B Fleming, Brandon T Pfannenstiel, Dianiris Luciano-Rosario, Harald Berger, Stefan Graessle, Mohamed M Alhussain, Joseph Strauss, Nancy P Keller, Koon Ho Wong, Özgür Bayram

**Affiliations:** Biology Department, Maynooth University, Maynooth, Co. Kildare, Ireland; Faculty of Health Sciences, University of Macau, Macau, China; Genomics, Bioinformatics and Single Cell Analysis Core, Faculty of Health Sciences, University of Macau, Macau, China; Biology Department, Maynooth University, Maynooth, Co. Kildare, Ireland; Faculty of Health Sciences, University of Macau, Macau, China; Faculty of Health Sciences, University of Macau, Macau, China; Faculty of Health Sciences, University of Macau, Macau, China; Genomics, Bioinformatics and Single Cell Analysis Core, Faculty of Health Sciences, University of Macau, Macau, China; Faculty of Health Sciences, University of Macau, Macau, China; Faculty of Health Sciences, University of Macau, Macau, China; Faculty of Health Sciences, University of Macau, Macau, China; Genomics, Bioinformatics and Single Cell Analysis Core, Faculty of Health Sciences, University of Macau, Macau, China; Biology Department, Maynooth University, Maynooth, Co. Kildare, Ireland; Institute of Molecular Biology, Biocenter, Medical University of Innsbruck, Innsbruck, Austria; Biology Department, Maynooth University, Maynooth, Co. Kildare, Ireland; Department of Microbiology, School of Genetics and Microbiology, Moyne Institute of Preventive Medicine, Trinity College Dublin, Dublin, Ireland; Department of Medical Microbiology and Immunology, University of Wisconsin, Madison, USA; Department of Medical Microbiology and Immunology, University of Wisconsin, Madison, USA; Department of Applied Genetics and Cell Biology, University of Natural Resources and Life Sciences, Tulln, Austria; Institute of Molecular Biology, Biocenter, Medical University of Innsbruck, Innsbruck, Austria; Department of Microbiology, School of Genetics and Microbiology, Moyne Institute of Preventive Medicine, Trinity College Dublin, Dublin, Ireland; Department of Applied Genetics and Cell Biology, University of Natural Resources and Life Sciences, Tulln, Austria; Department of Medical Microbiology and Immunology, University of Wisconsin, Madison, USA; Faculty of Health Sciences, University of Macau, Macau, China; Institute of Translational Medicine, Faculty of Health Sciences, University of Macau, Macau, China; Ministry of Education Frontiers Science Center for Precision Oncology, University of Macau, Macau, China; Biology Department, Maynooth University, Maynooth, Co. Kildare, Ireland

## Abstract

Chromatin complexes control a vast number of epigenetic developmental processes. Filamentous fungi present an important clade of microbes with poor understanding of underlying epigenetic mechanisms. Here, we describe a chromatin binding complex in the fungus *Aspergillus nidulans* composing of a H3K4 histone demethylase KdmB, a cohesin acetyltransferase (EcoA), a histone deacetylase (RpdA) and a histone reader/E3 ligase protein (SntB). *In vitro* and *in vivo* evidence demonstrate that this KERS complex is assembled from the EcoA-KdmB and SntB-RpdA heterodimers. KdmB and SntB play opposing roles in regulating the cellular levels and stability of EcoA, as KdmB prevents SntB-mediated degradation of EcoA. The KERS complex is recruited to transcription initiation start sites at active core promoters exerting promoter-specific transcriptional effects. Interestingly, deletion of any one of the KERS subunits results in a common negative effect on morphogenesis and production of secondary metabolites, molecules important for niche securement in filamentous fungi. Consequently, the entire mycotoxin sterigmatocystin gene cluster is downregulated and asexual development is reduced in the four KERS mutants. The elucidation of the recruitment of epigenetic regulators to chromatin via the KERS complex provides the first mechanistic, chromatin-based understanding of how development is connected with small molecule synthesis in fungi.

## INTRODUCTION

Eukaryotes feature complex and highly dynamic DNA-histone assemblies, including nucleosomes, tightly packed within the nucleus (1,2). Together with associated proteins, chromatin regulates all DNA-dependent processes such as transcription, replication, segregation and repair (3,4). Post-translational modifications (PTMs) such as acetylation, methylation and phosphorylation of the histone proteins determine which factors are recruited to or released from chromatin and are critical for the expression and, therefore, functionality of the underlying genetic information (5–7). To allow robust genome functions, chromatin states need to be highly dynamic and hence the deposition, recognition and removal of histone PTMs are tightly regulated processes in all eukaryotes (8).

Filamentous fungi represent a unique clade of eukaryotes that connect reproductive differentiation with secondary metabolite (SM) synthesis. Nearly 10 years ago, a pioneering study formally linked development and SM synthesis with the transcriptional Velvet complex in the fungus *Aspergillus nidulans* (9,10), a species long valued as a genetic model for both development (11) and SMs due to its synthesis of the carcinogenic SM sterigmatocystin (ST), the penultimate precursor to the deadly mycotoxin aflatoxin (12). A striking, conserved feature of SM biosynthetic pathways is their organization in physically linked 20–80 kb biosynthetic gene clusters (BGCs). Initial work with the ST BGC led to the finding that many BGCs are silenced by heterochromatic marks and hypo-acetylation in actively growing cells (13,14) or through methylation of histone H3 lysine 4 (15). Release of this chromatin-mediated repression is triggered by various environmental, nutritional or developmental signals and requires histone H3 and H4 acetylation by the SAGA-complex member GcnE (16) and EsaA (17), respectively. Extensive forward and reverse genetic analyses have revealed a number of additional epigenetic factors necessary for BGC activation such as the SANT-domain containing SntB chromatin reader (18) and the JmjC-domain containing H3K4me3 demethylase KdmB (19). Studies of SntB mutants also provided evidence for chromatin-based regulation of fungal development (20). However, it remains largely unclear how acetylases, methylases and other chromatin modifying factors are recruited to targets in filamentous fungi and if they act in parallel or in common complexes.

In order to approach this question we searched for interactors of KdmB and SntB using *in vivo* protein interaction strategies coupled with liquid chromatography-mass spectrometry (LC–MS/MS). Both proteins pulled down each other as well as the cohesin acetyl transferase EcoA, and the histone deacetylase RpdA, uncovering a multimeric complex that we name as KERS (KdmB-EcoA-RpdA-SntB). We found that KERS is assembled stepwise from EcoA-KdmB and SntB-RpdA heterodimers at the core promoter of >1600 target genes, enriched in regulatory genes controlling fungal development and synthesis of SMs including the carcinogen ST. This work advances our understanding of eukaryotic chromatin regulatory complexes and provides mechanistic explanations for previously scattered genetic evidence for the coupling of fungal development with chemical diversity.

## MATERIALS AND METHODS

### Strains, culture and growth conditions of *A. nidulans*

*A. nidulans* AGB551 was used as a host strain for genetic manipulations. Fungal strains were grown in GMM (glucose minimal medium): 1% d-glucose, 1× AspA (70 mM NaNO_3_, 7 mM KCl, 11.2 mM KH_2_PO_4_, pH 5.5), 2 mM MgSO_4_, 1× trace elements (76 μM ZnSO_4_, 178 μM H_3_BO_3_, 25 μM MnCl_2_, 18 μM FeSO_4_, 7.1 μM CoCl_2_, 6.4 μM CuSO_4_, 6.2 μM Na_2_MoO_4_, 174 μM EDTA). Complete media consisted of GMM with 0.1% yeast extract, 0.2% peptone, 0.1% tryptone and required supplements. For selective media, pyrithiamine (100 μg/ml) or nourseothricin (100 μg/ml) were added. Biotin (0.02 μg/ml), uracil (50 μg/ml), pyridoxine (0.05 μg/ml) were used as supplements when required. Culture and transformation procedures of *A. nidulans* and *Escherichia coli* used in this study were performed as previously described (21,22). For bacterial transformation, DH5α or MACH1 (ThermoFisher, Cat# C862003) *E. coli* competent strains were used to create recombinant plasmids as described previously (9). LB medium was supplemented with ampicillin (100 μg/ml).

### Plasmid and strain generations

Generation of all plasmids and strains has been described in detail in Supplemental Information. Oligonucleotides and plasmids used in this study are listed in Supplemental Table S1 and Supplemental Table S2. Strains used in this study are listed in Supplemental Table S3.

### Southern hybridization

Southern blot hybridization experiments were carried out using the Roche DIG Nucleic Acid Detection Kit (Roche, Cat# 11175041910). Amplification of either 5′ UTR or 3′ UTR regions (each yielding approximately 1.2 kbp) used as probes were carried out using the non-radioactive digoxigenin (DIG) labelling kit (Roche, Cat# 11093657910).

### Protein extraction, immunoblotting and antibody dilutions

Mycelia were lysed using lysis buffer B300: 50 mM Tris [pH 7.6], 300 mM NaCl, 1 mM EDTA, 0.1% NP-40, 10% glycerol, 1 mM dithiothreitol (DTT). The amount of proteins was measured by Bradford assay. 100 μg of total protein was loaded onto 4–15% precast SDS-PAGE gradient gels (Bio-Rad, Cat# 4561083) for whole cell-lysate analysis. For primary and secondary antibodies, the following conditions were used. 1:1000 dilution of mouse monoclonal α-Green Fluorescent Protein (GFP) (Santa Cruz, Cat# sc-9996); 1:1000 dilution of mouse monoclonal α-Hemagglutinin (HA) (Sigma, Cat# H9658). For detection of α-GFP and α-HA, 1:2000 dilution of Goat α-mouse (Bio-Rad, Cat# 1706516) was used. 1:2000 dilution of rabbit polyclonal α-SkpA (SconC) (custom made by Genscript, raised against peptide sequence EPIPIPNVSENVLSKVL), 1:2000 dilution of rabbit polyclonal α-Calmodulin Binding Protein (CBP) epitope tag (Sigma, Cat# 07-482), 1:2000 rabbit monoclonal (recombinant) α-acetyl-Lysine (Abcam, Cat# H9658). As secondary antibody, 1:2000 Goat α-Rabbit (Bio-Rad, Cat# ab190479) antibody was used. All antibodies were diluted in 5% non-fat milk in TBS (0.1% Tween-20), except for α-SkpA which required 0.5% Tween-20. Luminata Crescendo (Millipore, Cat# WBLUR0100) was used as a Western HRP substrate. For histone controls, 1 μg of core histones from Calf thymus (Sigma, Cat# 10223565001) were used. Membrane images were captured by a G-Box (Syngene). Membranes were stripped with stripping solution (0.2% Ponceau S Reagent, 3% TCA) overnight prior to detection of SkpA as a loading control.

### IgG Immobilization onto NHS-activated magnetic beads for Tandem affinity purification (TAP) coupled with liquid chromatography-mass spectrometry

5 mg of human IgG (Sigma, Cat# I4506) was dissolved in 3 ml Coupling Buffer (50 mM Borate, pH 8.5) and vortexed vigorously. 300 μl magnetic beads were added into 1.5 ml microcentrifuge tubes and supernatant was discarded using DynaMag™-2 Magnet (ThermoFisher, Cat# 12321D). Magnetic beads were washed with Wash Buffer A (1 ml of ice-cold 1 mM HCl) and vortexed gently for 15 sec. Then, supernatant was discarded using DynaMag™-2 Magnet. 300 μl protein solution (IgG in Coupling Buffer) was added to the magnetic beads and vortexed for 30 sec. Tubes were incubated at room temperature by rotation for 2 h, vortexed vigorously every 5 min for 15 s during the first 30 min of incubation. Then, tubes were vortexed every 15 min. At the end of 2 h of incubation, beads were collected and supernatant was saved into another tube for quality control. Samples were washed with 1 ml Wash Buffer B (0.1 M glycine, pH 2.0) and vortexed for 15 s. Beads were collected and supernatant was removed. This step was repeated one more time. Then, 1 ml ultrapure water was added to the beads and vortexed for 15 sec. Beads were collected and supernatant was discarded. 1 ml of Quenching Buffer was added to the beads, vortexed for 30 s and incubated at room temperature on a rotator for 2 h. Then, beads were collected and supernatant was removed. This step was repeated using ultrapure water and 1 ml Storage Buffer was added to the tubes and mixed well. Beads were collected and supernatant was discarded. This step was repeated two additional times. 300 μl storage buffer was added to the beads, mixed well and stored at 4 ºC for up to 6 months resulting in the final concentration of the IgG-coupled magnetic beads as 10 mg/ml IgG.

### Preparation of cell lysate & TAP tag protein purification

Preparation of buffers used in TAP method were as previously described (23). TAP-tagged fungal strains were inoculated in 800 ml liquid media and grown at 37ºC, 200 rpm for 24 h. Next day, mycelia were collected using miracloth and washed three times with harvest solution (PBS containing 100 μM PMSF and 1% DMSO). Remaining liquid was carefully removed by squeezing mycelia using paper towels prior to breaking mycelia down with the help of mortar/pestle in liquid nitrogen. Ground mycelia were immediately collected and stored in precooled 15 ml falcon tubes and kept in liquid nitrogen. SS34 tubes were pre-cooled for each strain and around 30 ml ground mycelia product was added to each tube. Complete Protease Inhibitor Cocktail EDTA-free (Roche, Cat# 11836170001), phosphatase inhibitors (Roche, Cat# 04906837001) and DTT were added to buffer B250 (100 mM Tris–HCl pH 7.6, 250 mM NaCl, 1 mM EDTA, 0.1% NP-40, 10% glycerol, 1 mM DTT). 12 ml of this buffer was added into each SS34 tube and vortexed vigorously. Tubes were kept on ice for about 10 min. At the end of mixing, tubes were centrifuged at 20 000 × g at 4 ºC for 20 min. During centrifugation, 15 ml falcon tubes for each sample were pre-cooled on ice. 50 μl of IgG-coupled NHS magnetic beads was added into a microfuge tube and 1 ml B250 buffer was added to wash the beads. Beads were collected and supernatant was discarded using DynaMag™-2 Magnet. Resuspended beads in 200 μl buffer were directly used for the next step. At the end of centrifugation, 12–15 ml supernatant from SS34 tubes was pipetted into pre-cooled falcon tubes and magnetic beads were added into the protein extract and incubated at 4 ºC for 3–4 h on a rotator. By the end of the incubation period, wash buffers (WB250, and WB150 with protease/phosphatase inhibitors, DTT and PMSF) were prepared. At the end of incubation, beads were collected and supernatant was discarded using DynaMag™-15 Magnet (ThermoFisher, Cat# 12301D) for 15 ml tube. 14 ml WB250 was added and mixed by inverting. Beads were collected and supernatant was discarded. This time beads were washed with 14 ml WB150. Next, 10 ml of TEV cleavage buffer (TCB, 50 mM Tris–HCl pH 8.0, 150 mM NaCl, 0.1% NP 40, 0.5 mM EDTA, 1 mM DTT and PMSF) was applied to each sample and mixed carefully. Beads were collected and supernatant was discarded. 1 ml TCB was added to resuspend the beads and solutions were transferred into new 1.5 ml microfuge tubes. 20 μl (200 U) TEV-protease (ThermoFisher, Cat# 12575015) was added to beads and incubated at 4ºC overnight on a rotating platform. Next day, CBB (25 mM Tris–HCl pH 8.0, 150 mM NaCl, 1 mM magnesium acetate, 1 mM imidazole, 2 mM CaCl_2_, 10 mM β-mercaptoethanol) was prepared by adding CaCl_2_ and β-mercaptoethanol prior to the calmodulin binding step. 50 μl MagnaZoom calmodulin magnetic beads (Bioworld, Cat# 20162002–1) was added to 1 ml CBB in 1.5 ml microfuge. Beads were washed and supernatant was discarded. Finally, beads were resuspended in 200 μl CBB. Supernatants from the TEV-treated samples were collected and were added directly into 6 ml of CBB in a 15 ml falcon tube as well as 7 μl of 1 M CaCl_2_. 200 μl CBB containing calmodulin magnetic beads were added into the same 15 ml falcon tube and incubated for 2–3 h at 4 ºC on a rotator. At the end of incubation, beads were collected and supernatant was removed using DynaMag™-15 Magnet. Beads were washed with 1 ml CBB and transferred into new 1.5 ml microfuge tubes. Next, beads were washed with 1 ml CBB, mixed well by inverting. Supernatant was discarded and this step was repeated 3 additional times. Protein bound beads were directly used for trypsin digestion in the next step.

### HA and GFP purifications

Strains were grown in GMM liquid cultures for 24 h at 37°C, 180 rpm. After harvesting and grinding, mycelia were lysed with lysis buffer supplemented with DTT, protease and phosphatase inhibitors. For protein extraction, lysis buffer (B300) was supplemented as follows: 150 μl 1 M DTT, 4 tablets Complete Protease Inhibitor Cocktail EDTA-free (Roche), 300 μl 0.5 M Benzamidine, 10 tablets phosphatase inhibitors (Roche) and 1 ml 100 mM PMSF, each per 100 ml of B300. Protein extracts and magnetic beads (20 μl of either GFP (Chromotek, Cat# Gtd-10) or HA paramagnetic beads (Pierce, Cat# 88836)) were incubated at 4°C for 2 h on a rotator prior to trypsin digestion. At the end of the incubation, beads were collected and supernatant was discarded using DynaMag™-Magnet. One ml of B300 (including 1 mM DTT) without protease and phosphatase inhibitors was added and mixed by inversion. Beads were collected and supernatant was discarded. This step was repeated three additional times. Protein bound beads were directly used for trypsin digestion in the next step.

### Trypsin digestion and sample preparation

Trypsin digestion for TAP, HA and GFP magnetic beads was performed as described in the manufacturer's protocol (ProteaseMAX, Promega, Cat# V5111). Trypsin-digested magnetic beads were discarded, peptides were precipitated, concentrated using speedy-vac and subjected to Zip-Tip C_18_ (Millipore, Cat# ZTC18S096) purification as described in manufacturer's protocol. Purified peptides were solubilized in Q-Exactive loading buffer (2% acetonitrile, 0.5% TFA in dH_2_O) and sonicated for 2 min.

### Proteomics analysis and peptide identification

Interaction partners of KERS complex and PTM identifications were analysed in at least two biological replicates filtered for unspecific peptides identified with AGB551 (untagged control strain). Protein samples were analysed using a Q-Exactive mass spectrometer coupled to a Dionex RSLCnano (Thermo Scientific). Peptides were separated using a 2–40% gradient of acetonitrile (A: 0.1% FA, B: 80% acetonitrile, 0.1% FA) over 65 min at a flow rate of 250 nl/min. The Q Exactive was operated in the data dependent mode, collecting a full MS scan from 300–1650 *m*/*z* at 70 K resolution and an AGC target of 1e^6^. The 10 most abundant ions per scan were selected for MS/MS at 17.5 K resolution and AGC target of 1e^5^ and intensity threshold of 1 K. Maximum fill times were 10 ms and 100 ms for MS and MS/MS scans respectively with a dynamic exclusion of 60 s. Samples were analysed using 25 NCE (normalized collisional energy) with 20% stepped energy.

Peptide identification using Proteome Discoverer 1.4 was performed using the Sequest HT (SEQUEST HT algorithm, Thermo Scientific) and searched against the UniProtKB-SwissProt database (taxonomy: *A. nidulans*). The following search parameters were used for protein identification: (i) peptide mass tolerance set to 10 ppm, (ii) MS/MS mass tolerance set to 0.02 Da, (iii) up to two missed cleavages were allowed, (iv) carbamidomethylation on Cys was specified as fixed modification (v) oxidation on Met and phosphorylation on Ser, Thr, Tyr were specified as variable modifications.

### Chromatin Immunoprecipitation (ChIP) sequencing and data analysis

ChIPseq experiments were performed at least two biological replicates. Crosslinking was performed with 1% formaldehyde at room temperature with gentle shaking for 20 min and subsequently was quenched by adding glycine to a final concentration of 125 mM. Mycelia was washed with water, harvested and pressed dried. Chromatin was prepared as described previously (24). Sonication was performed with Qsonica Q800R using 100% amplitude and 10 sec ON/OFF intervals for a total sonication time of 20 min. Immuno-precipitation was carried out as described previously (25,26) using 2 μl α-RNA polymerase II (Millipore, Cat# 04-1572), 2 μl α-HA (Santa Cruz, Cat# sc-7392), 10 μl α-myc (Santa Cruz, Cat# sc-40), 2 μg α-H3 (Abcam, Cat# ab1791), 2 μg α-H3K4me3 (Abcam, Cat# ab8580), 2 μg α-H3K9ac (Abcam, Cat# ab4441) or 2 μg α-H3K27ac (Abcam, Cat# ab4729). Immuno-precipitated DNA was subjected to library preparation for multiplex sequencing for Illumina sequencing according to (27). Libraries were mixed in equal molar ratio and sequenced using the Illumina HiSeq2500 platform. Sequencing reads were mapped to *Aspergillus nidulans* reference genome (AspGD version: s10-m04-r03) using Bowtie2 (v2.3.5) (28). Mapped reads were extended to 200 bp using ‘macs2 pileup’ command and scaled to 1 million mapped reads using ‘macs2 bdgopt’ command from MACS2 tool (29,30). UCSC Kent utils’ programs ‘bedSort’ and ‘bedGraphToBigWig’ were used to generate BigWig files (31). These normalized bigwig files were visualized on IGV, IGB and Pbrowse (32–34). Peak calling was carried out using MACS2 (v2.1.1) with parameters ‘–nomodel –extsize 200′ (29). KERS subunits’ binding signals were measured by counting the number of reads within 200 bp of peak summits and normalized to total read count of the dataset. KdmB binding signal was extracted for each gene using 2 kb upstream and 1 kb downstream regions and clustered using k-means clustering. ChIPseq against RNA polymerase II (Pol II) was performed for a WT strain grown under the same conditions as for the ChIPseq analysis of the complex members, and transcriptional activity of each gene was estimated by measuring Pol II occupancy over gene bodies (i.e. Pol II ChIPseq signal). Binding signal heatmaps were generated using Bioconductor package EnrichedHeatmap (v1.16.0) (35). Correlation analysis, GO analysis, Functional Network analysis (36) and heatmap plotting were performed on FungiExpresZ (Parsania and Wong, unpublished; https://cparsania.shinyapps.io/FungiExpresZ/).

### RNA extraction and Northern hybridization

RNA was extracted according to (37) as follows. Lyophilized mycelia of strains grown for 48 h in GMM supplemented with uracil/uridine and pyridoxine were ground to powder in a MixerMill (MM, Retsch, MM400) at 30 Hz for 3 min. Approximately 300 μl of mycelial powder was transferred to a 2 ml tube. After addition of 850 μl of NTES buffer (20 mM Tris–HCl pH 8.0, 100 mM NaCl, 10 mM EDTA pH 8.0, 1% (w/v) SDS) and 850 μl of phenol/chloroform/isoamylalcohol 25:24:1 (Carl Roth, Cat# A156.1) the tubes were thoroughly mixed in the MM at 30 Hz for 2 min and subsequently incubated at 65°C for 5 min. After centrifugation at 20 000 × g at 4°C for 10 min, the supernatant was transferred to a fresh tube and extracted with 0.3 vol. of PCI, mixed in the MM at 30 Hz for 20 s and centrifuged as above. PCI extraction was repeated and followed by an extraction with 0.3 vol. of chloroform. The resulting supernatant was mixed with 1 ml of TRI reagent and 200 μl of chloroform in the MM (30 Hz, 20 s). After centrifugation at 20 000 × g at 4°C for 10 min, the supernatant was divided onto two parts in 1.5 ml tubes and 250 μl of 1.2 M NaCl / 0.8 M Na citrate solution and 250 μl of isopropanol was added to each tube. The resulting precipitate was collected by centrifugation at 12 500 × g at RT for 15 min. The pellet was resuspended in 800 μl of 70% ethanol using a micropestle at 700 rpm. After centrifugation at 7500 rpm at RT for 10 min, the pellet was air-dried thoroughly and resuspended in 200 μl of nuclease-free water. Denaturing RNA electrophoresis, blotting, hybridization and detection were performed as described (38). Oligonucleotides used for the generation of Digoxigenin-dUTP-labeled DNA probes are listed in Supplementary Table S1.

### Protein alignment and ortholog analysis

Data was obtained using basic local alignment tool (BLAST; https://blast.ncbi.nlm.nih.gov/Blast.cgi) with *A. nidulans* KERS protein sequences as reference. Listed orthologs correspond to the proteins of highest scores obtained from the most significant alignments while values represent percentages of identification of protein sequence. EcoA homologs from human, yeast and Aspergillus were aligned using MegAlign Pro Clustal W alignment (DNASTAR, Lasergene 17.2)

### Confocal microscopy

Strains expressing sGFP and mRFP were grown in Lab-Tek chambered coverglass W/CVT (Thermo Scientific, Cat# 155360) in 400 μl GMM for 16 h at 30ºC. Microscopic images were captured as described earlier (39).

### Statistical analysis

Quantification of fungal development, sporulation and sexual fruit body formations were calculated using GraphPad Prism version 7 software. The level of significance was set at *P* < 0.05 (*), *P* < 0.01 (**), *P* < 0.001 and *P* < 0.0001 (****). Immunoblots were quantified using ImageJ (NIH) (40).

## RESULTS

### KdmB and SntB interact with EcoA and RpdA to form a conserved nuclear multi-domain complex

**Figure 1.**
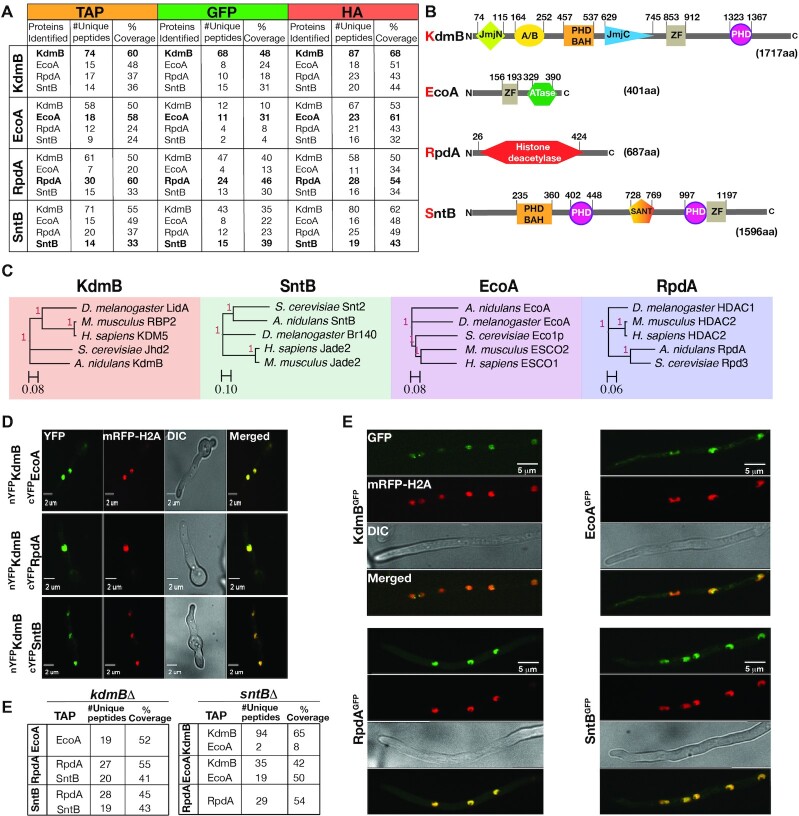
Discovery of the novel chromatin modifier KdmB-EcoA-RpdA-SntB (KERS) complex. (**A**) Selected interactors of KdmB, EcoA, RpdA and SntB, as identified with TAP/GFP/HA fusions by immunoprecipitation coupled with LC–MS/MS analysis. Fungal mycelia were harvested for immunoprecipitation at the end of 24 h vegetative culture at 37ºC. (**B**) Domain architecture of KERS complex members. PHD: plant homeodomain, ZF: zinc finger, A/B: arid/bright domain, BAH: Bromo-adjacent homology domain, SANT: Swi3, Ada2, N-Cor and TFIIIB domain, ATase: acetyltransferase. Numbers signify positions of the domains. (**C**) Orthologue analysis of the KERS complex among various organisms. KERS components are conserved from yeast to human. Trees were created using baker's yeast, *Aspergillus*, *Drosophila*, mouse and human homologs. (**D**) Bimolecular fluorescence complementation analysis with yellow fluorescent protein (YFP), verifying subcellular KdmB-EcoA, KdmB-RpdA and KdmB-SntB binary interactions. The images were captured by confocal microscopy at the end of 16 h incubation at 30 ºC under illumination. Nuclei were visualized in red by a monomeric red fluorescent protein fused to histone 2A (mRFP-H2A). (**E**) Cellular localization of KERS complex subunits. Confocal microscopy analysis of KdmB, EcoA, RpdA, SntB fused to GFP in a mRFP-H2A expressing strain. Strains were incubated and observed as above. (**F**) Identification of KERS subcomplexes in the absence of KdmB or SntB. LC–MS/MS analysis of EcoA, RpdA and SntB TAP purifications in *kdmB*Δ and *sntB*Δ genetic background, respectively. Growth conditions are the same as in (A).

KdmB and SntB were expressed as C-terminal functional fusions with TAP, GFP and HA tags from their original promoters at their native loci in separate strains (Supplementary Figure S1A, C and Supplementary Figure S2A). Potential KdmB and SntB *in vivo* interacting partners were identified by protein pull-down followed by LC–MS/MS. For each protein, two pull-down experiments were performed for the TAP-, GFP- and HA-tagged fusion independently, and eluates of all six pull-down material were subjected to LC–MS/MS (i.e. six independent repeats). Analysis of the combined results of the tagged fusions and the untagged control shows that both proteins interacted with each other as well as with the cohesin acetyltransferase EcoA and the histone deacetylase RpdA (Figure 1A). These interactions were confirmed by reciprocal EcoA and RpdA purifications and LC–MS/MS analysis (Supplemental Data S1–S12). Domain architectures of KdmB and SntB revealed that both proteins have the characteristic plant homeodomains (PHD) required for histone binding and chromatin-mediated gene regulation (Figure 1B). Basic Local Alignment Search Tool (BLASTp) using the protein sequence of the KERS subunits revealed that KdmB, EcoA, RpdA and SntB homologs are conserved from fungi to human (Figure 1C).

To consolidate the pull-down findings, we performed Bimolecular Fluorescence Complementation (BiFC) assays between KdmB and EcoA, RpdA and SntB and found that ^nYFP^KdmB fusion could interact with cYFP fusions of all three proteins (EcoA, RpdA and SntB) *in vivo* and that their interactions occurred within the nucleus (Figure 1D). In contrast, a control strain expressing ^nYFP^KdmB together with cYFP (without any fusion protein) showed no YFP signal (Supplementary Figure S1B). All strains showed 100% nuclear BIFC signal. Nuclear localization of the complex was further confirmed by confocal microscopy on strains expressing GFP fusions of the KERS complex with all KERS members (green color) co-localizing with monomeric red fluorescent protein-histone 2A fusion (mRFP-H2A, red color) (Figure 1E). Therefore, these results clearly demonstrated that KdmB, EcoA, RpdA and SntB form a stable core complex in the nucleus, which we named as KERS (KdmB-EcoA-RpdA-SntB). RpdA, KdmB and SntB have previously been shown to regulate histone PTMs (19,20,41), suggesting that KERS is a novel chromatin complex.

### The core KERS complex is assembled via association of EcoA-KdmB and SntB-RpdA subcomplexes

Chromatin modifying complexes often interact with scaffold proteins to target certain genomic regions (42). In order to analyse *in vivo* interactions within the KERS complex, TAP fusions of EcoA, RpdA and SntB in the *kdmB*Δ mutant were pulled down and analysed by LC–MS/MS. In the absence of KdmB, RpdA and SntB were not detected in the EcoA^TAP^ purification (Figure 1F, Supplemental Data S13) and conversely EcoA was not pulled down by RpdA^TAP^ and SntB^TAP^ purifications, while RpdA and SntB interaction was not affected (e.g. SntB^TAP^ pulled down RpdA and RpdA^TAP^ pulled down SntB) (Figure 1F, Supplemental Data S14 and S15). This suggests that the assembly of EcoA to the KERS complex depends on KdmB, while KdmB and EcoA are dispensable for RpdA and SntB heterodimer (SntB-RpdA) formation. On the other hand, similar pull-down experiments in the *sntB*Δ mutant background showed that EcoA and KdmB was able to form a heterodimer complex (e.g. KdmB^TAP^ pulled down EcoA and EcoA^TAP^ pulled down KdmB) (Supplemental Data S16-17), but neither KdmB nor EcoA were pulled down in the RpdA^TAP^ purification (Supplemental Data S18), suggesting that SntB is required for RpdA association with the EcoA–KdmB heterodimer for KERS formation. Taken together, these results demonstrate that KdmB and SntB act as essential scaffolds recruiting EcoA and RpdA, respectively, for the assembly of the KERS complex.

### KdmB prevents proteasomal degradation of EcoA mediated by SntB in KERS

**Figure 2.**
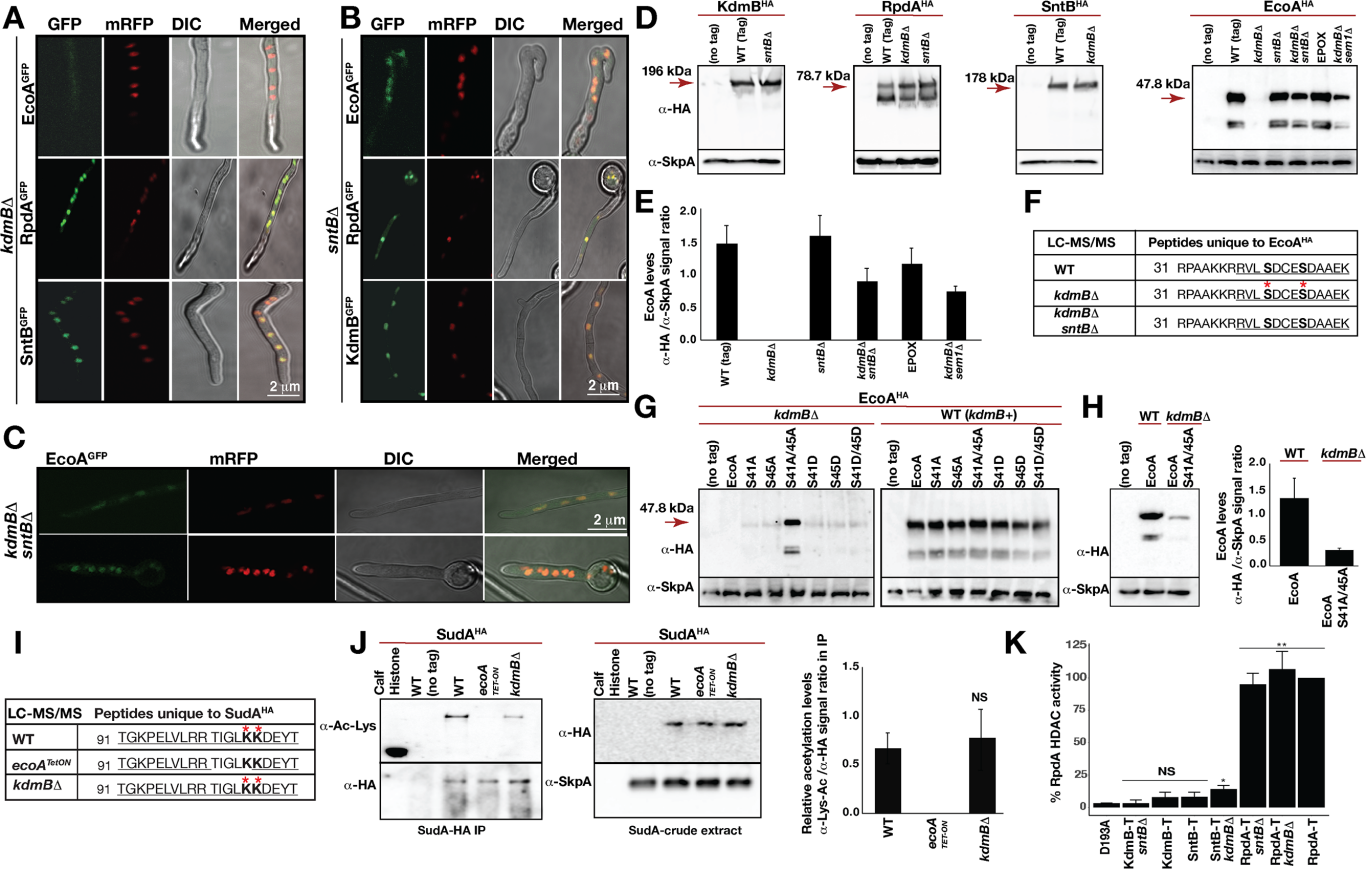
Cellular levels and enzymatic activities of KERS complex. Subcellular localizations of KERS GFP fusions in (**A**) *kdmB*, (**B**) *sntB* and (**C**) *kdmB/sntB* double mutants. (**D**) Cellular levels of KdmB-EcoA-RpdA-SntB in the WT and mutants. Fungal cultures were grown in submerged liquid GMM for 24 h at 37 ºC. α-HA monoclonal mouse and generic α-SkpA polyclonal rabbit antibodies were used to detect HA-fused protein and SkpA as a loading control, respectively. Crude extract (100 μg total protein) was used for immunodetection. Proteasome inhibitor epoxomicin (EPOX, 20 μM) was supplemented at the end of 20 h vegetative growth for a further 4 h of incubation. (**E**) Quantification of the EcoA^HA^ fusion in different backgrounds or treatments. EcoA^HA^/SkpA ratio was used for quantification in biological triplicates. (**F**) LC–MS/MS analysis of phosphorylated residues on EcoA in WT, *kdmB*Δ and in *kdmB*Δ*/sntB*Δ strains. The numbers indicate the position of amino acids. Red stars represent phosphorylated residues. Serines are depicted in bold. Underlined amino acids represent peptide coverage in MS analysis with high confidence. (**G**) Expression of EcoA^HA^ and its mutant versions of S41A, S45A, S41A/S45A, S41D, S45D, S41D/S45D in *kdmB*Δ and WT. (**H**) Comparison of expression levels of EcoA^HA^ and S41A/S45A mutant of EcoA^HA^ left (blot) and right (quantification) from three biological triplicates. (**I**) Acetylation of SudA^K106/107^ (yeast Smc3 homolog) cohesin by EcoA and loss of acetylation in *ecoA^TetON^*. Red stars and bold letters represent acetylated residues. Kit-enriched acetylated peptides from immunoprecipitation of SudA^HA^ were analysed in LC–MS/MS. (**J**) Verification of LC–MS/MS acetylation levels of immunoprecipitated SudA^HA^ with Lys-Acetylation specific antibodies. α-Lys-Ac/α-HA signal ratio was used for quantification of acetylation from four biological replicates. (**K**) *In vitro* histone deacetylase activity (HDAC) of purified KERS complexes. T represents TAP of the corresponding proteins in WT and mutants. Enzymatic activity of enriched RpdA complexes was measured in triplicates using [H-3]-acetate prelabelled chicken histones (52). Blank levels were subtracted and all samples were calculated with respect to RpdA-TAP activity adjusted to 100%. HDAC activity assay is the average of three independent biological replicates. Significance relative to D193A as calculated by Student's t-test is indicated as asterisks: *P* < 0.05 (*) and *P* < 0.01 (**).

To investigate the interdependence of the KERS subunits for nuclear localization, all complex components were expressed as GFP fusions in the absence of KdmB, SntB or both (Figure 2A–C). RpdA^GFP^ and SntB^GFP^ localizations were not affected in the absence of KdmB (Figure 2A). Similarly, KdmB^GFP^ and RpdA^GFP^ localizations were not influenced by the loss of SntB (Figure 2B). Interestingly, while *sntB* deletion did not have any effect on EcoA nuclear localization, EcoA^GFP^ could not be detected in the *kdmB*Δ mutant (Figure 2A). This result may be due to KdmB’s control on EcoA nuclear localization and/or EcoA protein level. To address this, it was investigated whether the cellular level of EcoA was dependent on KdmB. Western blot analysis showed that EcoA protein level was dramatically reduced (by ∼95%) in the *kdmB*Δ mutant compared to WT (Figure 2D, E and Supplementary Figure S2B). In contrast, the EcoA level was unaffected in the *sntB*Δ strain, and expression of the other KERS subunits (KdmB, SntB and RpdA) was also not affected in the *kdmB*Δ or *sntB*Δ mutants (Figure 2D, E). Interestingly, loss of EcoA in the *kdmB*Δ mutant was not reflected transcriptionally, as *ecoA* mRNA levels were similar in WT, *kdmB*Δ, *sntB*Δ and in *kdmB*Δ*/sntB*Δ strains (Supplementary Figure S2C), suggesting that the observed reduction in EcoA is likely due to a post-transcriptional regulation.

In yeast, Eco1 is prone to proteasomal degradation (43) and Snt2, the homologue of SntB, has a E3 ubiquitin ligase activity (44). However, a functional and physical connection between Snt2 and Eco1 degradation has not been documented. We hypothesized that in *A. nidulans* SntB may promote the degradation of EcoA while KdmB protects EcoA from degradation. Consistent with this hypothesis, deletion of *sntB* in the *kdmB*Δ background (i.e. *kdmB*Δ*/sntB*Δ double mutant) partially restored (∼60%) EcoA protein levels (Figure 2D–E) as well as its nuclear localization (Figure 2C). Most importantly, treatment of the *kdmB*Δ mutant with the inhibitor of proteasome epoxomicin (45) or deletion of *sem1* (a subunit of proteasome (46)) in the *kdmB*Δ mutant prevented EcoA from degradation (Figure 2D). Overall, these data revealed a novel interplay between KdmB-SntB on proteasomal degradation of EcoA.

### Phosphorylation of the Ser41 and Ser45 residues of EcoA promotes its degradation in the absence of KdmB

It was of interest to determine the mechanism behind the KdmB-SntB interplay on EcoA degradation. KdmB protection of EcoA from SntB-dependent degradation may be mediated by physically blocking the SntB-EcoA interaction. On the other hand, a direct KdmB interaction with EcoA may control PTM(s) of EcoA. In yeast, degradation of Eco1 is initiated on phosphorylation of Ser 98, Ser 99 and Thr 94 by Cdk1, Dbf4-Cdc7 and Mck1, respectively, and consequently leading to ubiquitination of Eco1 by SCF^Cdc4^ (43,47,48). Of note, the reported phosphorylation sites (Ser 98 and 99) of Eco1 are not present in *A. nidulans* EcoA (Supplementary Figure S3). To determine if EcoA degradation is regulated by phosphorylation and to identify the phosphorylation events involved, LC–MS/MS analysis was performed on EcoA^HA^ purified from WT, *kdmB*Δ and *kdmB*Δ/*sntB*Δ strains employing a phosphorylation-sensitive method. The result showed that EcoA^S41/S45^ residues were specifically phosphorylated when KdmB was absent but not in wildtype (WT) and the *kdmB/sntB* double mutant (Figure 2F), suggesting that phosphorylation of these two sites is responsible for the degradation observed in the *kdmB*Δ mutant. These phosphorylation sites are located at the N-terminal end of EcoA that do not exist in yeast Eco1, which is relatively shorter at the N-terminus (Supplementary Figure S3).

We generated phosphomimetic and non-phosphorylatable EcoA mutants by mutating the two Ser residues individually or in combination to either Aspartate or Alanine, respectively, in WT and the *kdmB*Δ mutant. All of the mutant strains were phenotypically similar to WT (Supplementary Figure S4). Consistent with the LC–MS/MS findings, simultaneous mutation of both Ser residues to Ala (S41A/S45A) resulted in stabilization of EcoA in the *kdmB*Δ mutant, while mutation of either serine residue (S41A or S45A) was not sufficient for the stabilization (left blot, Figure 2G). Of note, the stabilized amount of the non-phosphorylatable EcoA^S41A/S45A^ in the *kdmB*Δ mutant restored ∼15–20% of the WT EcoA level (Figure 2H). This result indicates the existence of additional phosphorylation site(s) and/or an additional control other than EcoA^S41/S45^ phosphorylation. Alternatively, the interaction of KdmB and EcoA alone may be sufficient to protect EcoA from degradation by the proteasome. In support of the latter possibility, none of the phosphomimetic EcoA mutations (either singly or in combination) could induce EcoA degradation in WT where KdmB dimerizes with EcoA in the KERS complex (right blot, Figure 2G). Taken together, the above results demonstrate that KdmB protects EcoA from phosphorylation at the EcoA^S41/45^ residues and SntB-mediated proteasomal degradation.

### EcoA-mediated acetylation of the cohesin SudA is unaffected in the *kdmB*Δ mutant

In yeast, Eco1 acetylates cohesin Smc3 subunit, which is essential for cell cycle and chromosome stability (49). Expression and LC–MS/MS of *A. nidulans* cohesin subunit SudA^HA^ fusion in WT and the *kdmB*Δ and *ecoA^TetON^* mutants showed that two lysine residues, SudA^K106/K107^, were acetylated by EcoA (Figure 2I). This acetylation disappeared in *ecoA^TetON^* strain in the absence of doxycycline, which shuts down the Tet-ON promoter. Lack of KdmB does not influence acetylation in SudA^K106/K107^ residues. The findings of LC–MS/MS was further corroborated by Lys acetylation specific antibodies which detected acetylated Lys residues in immunoprecipitated SudA^HA^ (Figure 2J) in relevant background strains, suggesting that the small fraction (∼5%, Supplementary Figure S2B) of full length EcoA detected in the *kdmB*Δ strain is sufficient for full SudA acetylation. Although it is formally possible that an EcoA-related protein may be responsible for SudA acetylation, based on BLAST analysis, EcoA is the only candidate with significant homology to yeast Eco1 and human ESCO1/ESCO2.

### RpdA HDAC activity is inhibited by association with the KERS complex

SANT domain-containing proteins such as yeast Snt2 (SntB, Figure 1B) have been shown to promote Rpd3 HDAC activity (50). The above Proteomic analysis showed that RpdA is part of the KERS complex recruited by SntB. This result raises an important question of whether RpdA HDAC activity is influenced by association with SntB and/or the KERS complex. To elucidate this, an *in vitro* HDAC activity assay was performed as described previously (51). IgG pull-downs using SntB or KdmB as bait did not display significant HDAC activity compared to a catalytically inactivated (D193A) RpdA version (Figure 2K) (52). *kdmB*Δ significantly raised HDAC activity of the remaining SntB-RpdA complex, whereas no HDAC activity was detected in the KdmB pull-down of an *sntB*Δ strain (KdmB-T-*sntB*Δ; Figure 2K). The latter observation is in line with the fact that RpdA is recruited to the KERS complex by SntB (Figure 1F). HDAC activity in pull-downs using RpdA as bait was generally much higher regardless of whether KdmB or SntB functions are present (RpdA-T, RpdA-T *kdmB*Δ, RpdA-T *sntB*Δ; Figure 2K), presumably due to the purification of additional RpdA complexes such as RpdA-L, RpdA-S and RcLS2F (38) that are independent of KERS. Overall, these *in vitro* evidence provides additional independent supports for the dependence of SntB for RpdA recruitment. More importantly, the results suggest an inhibition of the HDAC activity of RpdA when it is associated with the KERS complex but not when it is in the heterodimeric SntB-RpdA subcomplex.

### KERS functions to recruit its member subunits to the pre-initiation complex at the core promoter of active genes

**Figure 3.**
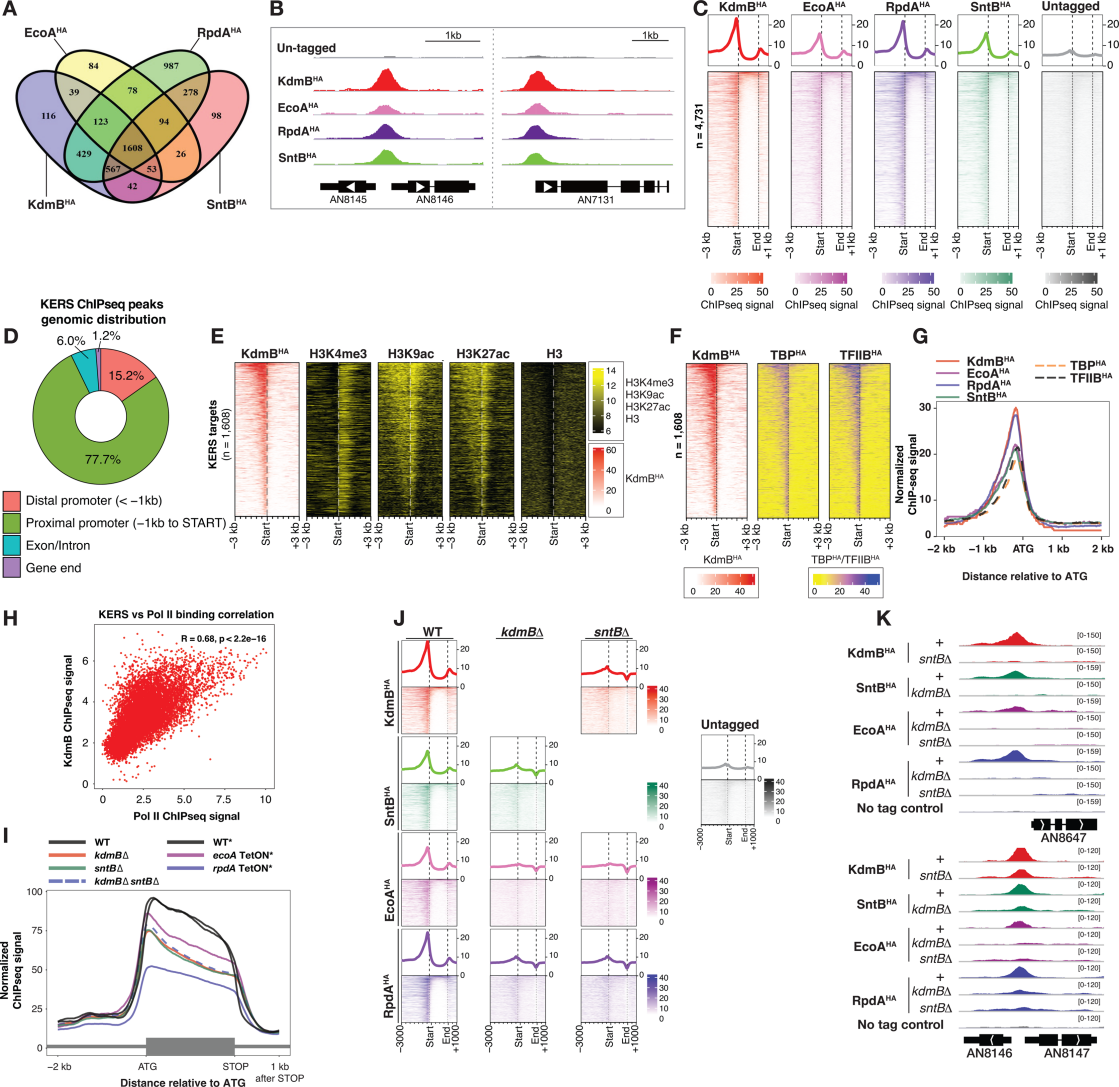
KdmB, EcoA, RpdA and SntB co-localized at thousands of promoters (**A**) A VENN diagram showing the number of common and unique genes between KdmB^HA^, EcoA^HA^, RpdA^HA^ and SntB^HA^. (**B**) Genome-browser screenshots showing the binding location of KdmB^HA^, EcoA^HA^, RpdA^HA^ and SntB^HA^. (**C**) Heatmaps displaying binding locations, signals and intensities of KdmB^HA^, EcoA^HA^, RpdA^HA^ and SntB^HA^ over their overall target genes (*n* = 4165). (**D**) A pie chart showing the distribution of the common KERS binding sites at distal promoters, proximal promoters, genic regions (exon/intron) and gene ends. (E, F) Heatmaps displaying ChIPseq signals of (**E**) KdmB^HA^, histone H3 lysine 4 trimethylation (H3K4me3), histone H3 lysine 9 acetylation (H3K9ac), histone H3 lysine 27 acetylation, histone H3, (**F**) TBP^HA^ and TFIIB^HA^ at the –3 kb to + 3 kb region with respect to the start codon (ATG) of KERS target genes. (**G**) A line graph showing the median binding signals of KdmB^HA^, EcoA^HA^, RpdA^HA^, SntB^HA^, TBP^HA^ and TFIIB^HA^ at the –2 kb to + 2 kb region with respect to the start codon (ATG) of KERS target genes. (**H**) A scatter plot showing the correlation between KdmB bindings at promoters and Pol II occupancies at coding regions. (**I**) A line plot showing the median Pol II ChIPseq signals between 2 kb upstream (–2 kb) of the translational start codon (ATG) to 1 kb downstream (+1 kb) of the translational stop codon of expressed genes in the WT and mutant strains deleted (Δ) or depleted (*TetON*) for the indicated subunit. The asterisk (*) indicates strains grown under the same experimental conditions for protein expression shutdown by the TetON system. (**J**) Heatmap plots showing binding locations, signals and intensities of KdmB^HA^, EcoA^HA^, RpdA^HA^ and SntB^HA^ in WT, *kdmB*Δ or *sntB*Δ background. (**K**) Genome-browser screenshots showing binding of KdmB^HA^, EcoA^HA^, RpdA^HA^ and SntB^HA^ in WT (+), *kdmB*Δ or *sntB*Δ background.

The nuclear localized KERS subunits bear chromatin binding domains and are expected to function on the chromatin. However, the four subunits have distinct enzymatic activities and substrate specificities. We next investigated whether the four subunits are recruited as a complex and function at the same chromatin locations using Chromatin Immuno-precipitation followed by Next Generation Sequencing (ChIPseq) analysis. Peak-calling analysis by MACS2 identified a large number of binding sites for each of the four proteins with EcoA^HA^ (*n* = 2412) having the least binding sites. On the other hand, KdmB^HA^ and SntB^HA^ have roughly the same number of binding sites (*n* = 3163 and 2915, respectively), while RpdA^HA^ has the most bindings (*n* = 4165) and many of them are not bound by the other members of the KERS complex (Figure 3A and Supplemental Data S19). Inspection of the data on Genome-browser showed that the four proteins bind to the same sites at many gene promoters, as illustrated by the bindings at the three randomly selected representative genes in Figure 3B. Systematic analysis identified 1,608 genomic regions commonly bound by all four KERS subunits (Supplemental Data S20). More importantly, the proteins share similar binding profiles (Figure 3C and Supplementary Figure S5), supporting the above findings that KERS components interact and form a core complex.

Unexpectedly, the majority of these promoter bindings appear as point source peaks at promoter regions similar to those observed for transcription factor bindings, with only a small percentage of coding regions showing broad occupancy by the complex (Figure 3C, D). The result was unexpected because KdmB is a H3K4me3 demethylase and therefore, expected to co-localize (at least partially) with H3K4me3 modification, which was shown to be enriched over a broad region near the 5′ end of active genes in *A. nidulans* and *A. fumigatus* (53,54). This prompted us to compare KERS genomic occupancy with H3K4me3 modifications (mapped by ChIPseq) in WT *A. nidulans*. Surprisingly, there is little overlap between KERS occupancy (at core promoter regions) and H3K4me3 modification (near the 5′ end of coding region of active genes) (Figure 3E and Supplementary Figure S6A), implying that the KdmB demethylase is not directly recruited to H3K4me3-modified nucleosomes. Moreover, those KERS-occupied promoters are also not devoid of histone acetylation (e.g. H3K9ac and H3K27ac; Figure 3E and Supplementary Figure S6A), which would be the case if the RpdA subunit of the KERS complex were active. Therefore, these results indicate that the observed KERS promoter occupancies is marking the sites of KERS recruitment and assembly rather than the locations where KERS exerts its chromatin modifying function(s), presumably the interactions of the complex and/or individual subunits with their active chromatin sites (i.e. H3K4me3-modified nucleosomes at the body of active genes to be demethylated) are too transient for detection by the ChIP technique (55,56). Nevertheless, the ChIPseq results revealed the genes targeted by the KERS complex under the experimental conditions analysed.

The genomic location of the observed KERS bindings suggest that the KERS complex is recruited to gene promoters. Of note, the regions showing highest ChIPseq signal for KERS correspond to the nucleosome-free region at core promoters (as indicated by the relatively lower levels of H3 ChIPseq signals in Figure 3E), which are bound by transcription factors and where Pol II transcription initiates. We performed MEME-ChIP analysis on the summits of KERS common binding sites to identify transcription factor(s) recruiting the complex (57). Interestingly, many motifs were enriched (Supplementary Table S4) including the consensus motif of well-established transcription factors such as the CCAAT-binding complex (CBC) (58) and TBF1, which are transcriptional regulators of metabolism and ribosomal protein genes, respectively, that are highly expressed under the experimental conditions analysed.

The enrichment of many motifs among KERS binding sites suggests a general role of the KERS complex (i.e. KERS is not recruited by specific transcription factors). Therefore, we next determined whether the KERS complex is associated with the general transcription factors at the transcription pre-initiation complex (PIC) by comparing KERS binding profile with those of TBP^myc^ and TFIIB^myc^ (53). Indeed, the binding location and the ChIPseq peak shape of the KERS complex are indistinguishable from those of TBP and TFIIB (Figure 3F, G and Supplementary Figure S6B). In agreement with these findings, KdmB^TAP^ pull-down copurified TBP (Supplemental Data S1). More importantly, there is a positive correlation (R = 0.68) between genome-wide KERS bindings and transcription of respective genes as measured by active RNA polymerase II (Pol II) ChIPseq (25) (Figure 3H and Supplemental Data S21), and the correlation level is similar to those of TBP and TFIIB with Pol II as reported previously (*R* = 0.652 and 0.525, respectively) (53). Therefore, KERS bindings at promoters positively correlate with PIC and Pol II transcription levels as well as with the active chromatin marks H3K4me3, H3K9ac and H3K27ac (Figure 3E). The positive correlations are inconsistent with the established role of KdmB and RpdA in removing histone marks associated with active transcription (19) (i.e. they play a repressive role), further supporting the observed KERS binding events being the recruitment rather than operation sites. The overall observations suggest that KERS is universally recruited and assembled at the PIC of active genes.

### The KERS complex does not affect the actual transcription process

The binding of KERS at core promoters overlapping the PIC suggests a possible role of the complex in controlling the actual transcription process (e.g. transcription initiation, elongation and termination). To address this, we determined the occupancy profile of Pol II captured by ChIPseq, which we have previously used to identify defects in the transcription process in *S. cerevisiae* (55,59,60). The Pol II occupancy profiles of WT and the KERS mutants that fail to assemble the complex at core promoters are indistinguishable (Figure 3I), indicating that the assembly and binding of KERS at core promoters do not affect the overall transcription process *per se*.

### The assembly of the full KERS complex is essential for its recruitment to and stable association at core promoters

The above biochemical experiments demonstrated essential roles of KdmB and SntB in the formation of the core KERS complex by recruiting EcoA and RpdA through KdmB-EcoA and SntB-RpdA dimers (Figure 1F). We next addressed whether KdmB and SntB are also essential for the recruitment of the KERS complex to PIC. This is indeed the case as shown by ChIPseq analysis of individual KERS complex members in the *kdmB*Δ or *sntB*Δ mutants under the same growth conditions. When compared to WT, chromatin bindings by all members are drastically affected in the *kdmB*Δ and *sntB*Δ mutants (Figure 3J, K). The lack of binding in the mutants is not due to an effect in the expression of the complex members as shown by Pol II ChIPseq and western blot analysis (Supplementary Figure S7A and Figure 2D), except for EcoA that is degraded in the *kdmB*Δ mutant. Therefore, the pull-down and ChIPseq results demonstrate that stable association of the KERS complex to the chromatin requires formation of intact dimers and the tetrameric complex. Overall, the results indicate a mechanism for the KERS complex in recruiting the histone demethylase KdmB, the histone deacetylase RpdA, the cohesin acetyltransferase EcoA and the chromatin reader SntB to the PIC of active genes.

### The four subunits of KERS have distinct transcriptional effects

**Figure 4.**
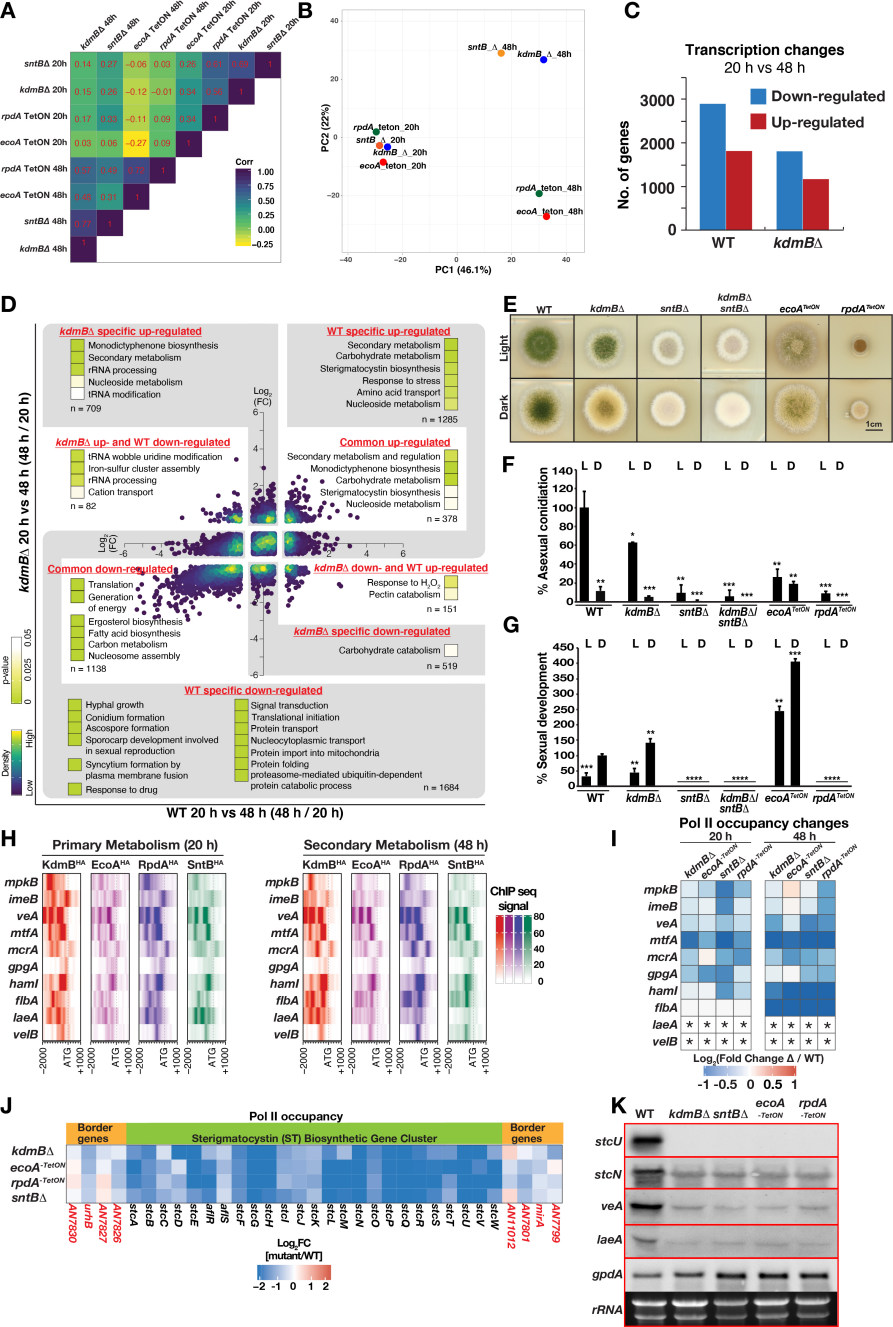
The KERS complex regulates diverse physiological pathways in development and the carcinogen sterigmatocystin biosynthesis. (**A**) A heat box plot showing the correlations of transcriptional effects (measured by Pol II ChIPseq signals at coding regions in the respective mutant versus WT) between the KERS mutants grown under primary (20 h) and secondary (48 h) growth phases. (**B**) PCA analysis on the transcriptional effects (measured by Pol II ChIPseq signals at coding regions in the respective mutant versus WT) of the KERS mutants grown under primary (20 h) and secondary (48 h) growth phases. (**C**) The number of up- and down- regulated genes (Pol II ChIPseq) during transition from primary (20 h) to secondary (48 h) growth phase in WT and the *kdmB*Δ mutant. (**D**) An overview of GO enrichment analysis for genes whose response to primary-to-secondary growth phase transition differs between WT and the *kdmB*Δ mutant. (**E**) Functions of the KERS complex components on fungal development and light responses. Growth of KERS mutants (deletions or *TetON* strains) on GMM plates at 37 ºC for 5 days both under continuous white light and dark conditions. (**F, G**) Quantification of asexual (conidia) and sexual (fruiting bodies) development from (**E**). Significance as calculated by Student's t-test is indicated as asterisks: *P* < 0.05 (*), *P* < 0.01 (**), *P* < 0.001 and *P* < 0.0001 (****). Asexual sporulation and sexual development were calculated relative to WT-light conidiation and WT-dark sexual development, respectively. (**H**) Heatmaps displaying binding locations, signals and intensities of KdmB^HA^, EcoA^HA^, RpdA^HA^ and SntB^HA^ over regulatory genes of the ST BGC and development. (**I**) A heatmap showing expression level of genes associated with positive regulation of SM biosynthesis in the KERS mutants at primary and secondary growth phases. Expression is presented as log_2_ fold change with respect to values in WT. Asterisks (*) indicate non-detectable Pol II ChIPseq signal in both WT and mutant strains, and hence fold change cannot be calculated. (**J**) A heatmap showing expression (as Pol II ChIPseq) levels of the ST BGC and border genes at secondary growth phase. (**K)** Validation of a few key genes from (**H**) by Northern blotting. Strains were grown for 48 h (Secondary Metabolism) in GMM. 10 μg of total RNA was used per lane. rRNA signals and *gpdA* served as loading control.

We next sought to determine whether the subunits of KERS functioning together as a whole complex or whether the KERS core complex only serve as a recruitment platform with the different subunits playing promoter-specific (i.e. independent) roles by comparing the transcriptional effects in the four individual mutants. Similar transcriptional effects are expected for strains lacking one of the four mutants if KERS acts together for the same function. On the other hand, the transcriptional effects would be significantly different if the KERS complex merely serves to recruiting the subunits to active core promoters. Genome-wide transcription activities in WT and the mutants were measured under primary (i.e. actively growing) and secondary (i.e. stationary) growth phases (at 20 and 48 h post-inoculation, respectively) using Pol II ChIPseq, which is a powerful method for capturing dynamic transcription changes (25). Correlation analysis shows that all four mutants have significantly different transcriptional effects (i.e. poor correlation) under both primary and secondary growth phases when comparing to WT (Figure 4A). Interestingly, PCA analysis revealed that the *kdmB*Δ and *sntB*Δ mutants have relatively similar transcriptional effects under secondary growth phase, while the loss of RpdA and EcoA produce yet another distinct but related transcriptional effects (Figure 4B). This is in agreement with the scaffolding roles of KdmB and SntB in establishment (Figure 1F) and chromatin recruitment of the KERS complex (Figure 3J, K) results. Taken together, these observations suggest that the subunits have independent functions and, therefore, the primary function of the core KERS complex is for chromatin recruitment of the epigenetic regulators.

### KERS is required for the transcriptional blueprint directing diverse physiological processes including sexual and asexual development and secondary metabolism

In WT, transition from primary to secondary growth phases was accompanied with up- and down-regulation of almost 5000 genes (Figure 4C and Supplemental Data S22). Genes enriched in the physiological processes such as translation, generation of precursor metabolites and energy, hyphal growth and signal transduction, *etc*. were down-regulated, while genes involved in amino acid transport, response to stress as well as carbohydrate, nucleoside and secondary metabolism were significantly up-regulated (Supplementary Figure S7B, C). In contrast to WT, only ∼3000 genes were differentially expressed in the *kdmB*Δ mutant (Figure 4C and Supplemental Data S22).

Gene ontology analysis of WT-specific regulated genes (i.e. those not significantly changed in the *kdmB*Δ strain) revealed defects of the *kdmB*Δ strain in down-regulating genes required for hyphal growth, translational initiation, signal transduction and developmental processes among others as well as in secondary, carbohydrate and nucleoside metabolism genes (Figure 4D and Supplemental Data S23). More importantly, some of these affected physiological processes are also targeted by all four proteins of the KERS complex as described above (Supplementary Figure S7). For example, many genes involved in asexual and sexual development such as asexual regulators *flbA*, *flbD* and sexual regulators *nsdD*, *nsdC*, *steA*, *csnC* (a COP9 signalosome subunit) (61) were bound by KERS and mis-regulated in the mutants (Figure 4D and Supplementary Figure S8).

To confirm the physiological significance of KERS on fungal development, which is directed by light regimes where white light promotes asexual sporulation and absence of light promotes sexual development, we assessed the growth, and asexual and sexual phenotypes of *kdmB* and/or *sntB* deletion mutants as well as of strains expressing the essential *ecoA* or *rpdA* genes under a conditional TetON promoter. Only *rpdA^TetON^* strain showed drastic reduction in radial growth, consistent with a previous study (41). Interestingly, light-dependent asexual sporulation was reduced in *kdmB*Δ, *ecoA^TetON^*, *rpdA^TetON^*and *sntB*Δ strains by 40–90% (Figure 4E, F). On the other hand, sexual development was eliminated in the *sntB*Δ and *rpdA^TetON^*strains, but was stimulated in the *kdmB*Δ and *ecoA^TetON^* mutants (Figure 4G). This suggested balanced roles of the KdmB-EcoA and RpdA-SntB heterodimers in regulating sexual development. Strikingly, *sntB*Δ was epistatic to *kdmB*Δ for sexual development as evident from the phenotype of the *sntB/kdmB* double mutant. Together, the data demonstrate a strong role for KERS in light-dependent fungal development.

Considering that one of our original motivations for initiating this study stemmed from our interest in elucidating mechanisms regulating the carcinogen ST, we examined expression of all genes known to positively and negatively regulate this toxin. For example, eight out of ten genes associated with regulation of the toxin ST were bound by the full KERS complex under both primary (20 h) and secondary metabolite (48 h) growth conditions (Figure 4H) and showed decreased transcription (measured by ChIP Pol II ChIPseq) in almost all KERS mutants (Figure 4I). These ten regulatory genes included two mitogen-activated kinases (MAPK), *mpkB* (39,62) and *imeB* (63), the components of the velvet transcriptional complex (9), *veA*, *velB* and *laeA*, the C_2_H_2_ zinc finger *mtfA* (64), a global transcription factor of SMs *mcrA* (65) and the heterotrimeric gamma subunit *gpgA* (66), and regulators of hyphal fusion, *hamI*, as well as the conidiation regulator *flbA* (18,67) (Supplemental Data S23). We also assessed the transcriptional response of the ST BGC to all four proteins of the KERS complex and found that the entire ST BGC was transcriptionally down regulated in all four mutants of the KERS complex consistent with the reduction of ST production in *kdmB*, *sntB* and *rpdA* mutants after 48 h of growth (18,19,68) (Figure 4J). The transcriptional changes of *veA* and *laeA* which are two key regulators of secondary metabolism and development and ST genes (*stcU* and *stcN*) in the mutant detected by Pol II ChIPseq were validated by Northern blot analysis after 48 h of growth (Figure 4K). Taken together, our results reveal a mechanistic link between morphological differentiation and secondary metabolism via the novel KERS complex identified in this work.

## DISCUSSION

**Figure 5.**
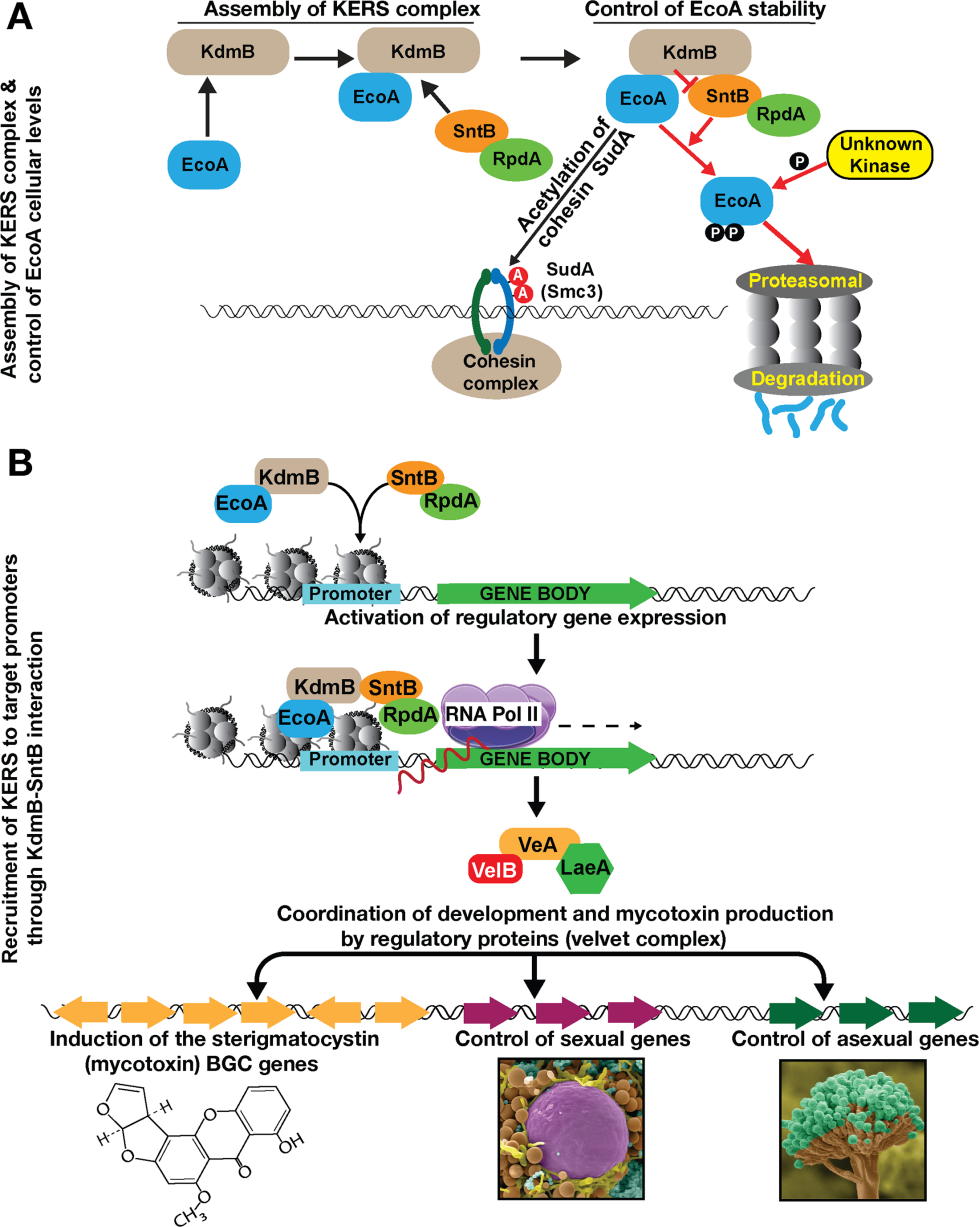
Chromatin level coordination of fungal development and mycotoxin production by the KERS complex. (**A**) Molecular mechanisms of KERS assembly. Two heterodimers, KdmB-EcoA, SntB-RpdA establish the KERS core complex. EcoA acetylates the cohesin SudA at two residues. KdmB protects EcoA from proteasomal degradation by preventing its phosphorylation and subsequent ubiquitination presumably by the ring finger E3 ligase SntB. (**B**) The KERS binds to more than 1,600 promoters including many regulatory target genes and activates the transcription of these genes. These regulatory genes, particularly, velvet complex genes whose expression depends on the KERS complex, control production of mycotoxin and its coordination with fungal development.

Modulation of gene expression by multimeric chromatin complexes is one of the fundamental regulatory mechanisms in eukaryotes. We have identified a novel chromatin-binding core complex, termed KERS, that assembles via two heterodimers of EcoA-KdmB and SntB-RpdA (Figure 5A) and binds to more than 1,600 promoters in the fungal genome during different physiological conditions (Figure 5B). Both KdmB and SntB control cellular levels of EcoA through proteasomal degradation (Figure 5A). Gene ontology analysis of binding sites revealed an overrepresentation of regulatory genes involved in cell signalling and transcriptional regulation, some of which are regulators of fungal development and SM production. These regulatory pathways then coordinate development and SM production (Figure 5B). Particularly, the velvet complex, which coordinates SM and development, represents one of these regulatory targets. The two key subunits of the complex, VeA and LaeA (9,69), are downregulated in all KERS mutants.

Filamentous fungi represent a unique group of organisms where developmental processes are inextricably tied to chemical development. Each species contains dozens of BGCs and extensive studies have convincingly shown that many BGC products provide either protection from abiotic (e.g. UV radiation) or biotic (e.g. microbial competition) stresses (70,71). Timing of BGC activation can be critical, such as synthesis of the asexual spore melanin BGC during spore development (72) or induction of antibacterial BGCs during competition with bacteria (73). Because filamentous fungi make up the majority of pathogenic fungi and their BGC products are both harmful (e.g. mycotoxins) or beneficial (e.g. pharmaceuticals), there has been tremendous interest in understanding how BGCs are regulated and how this regulation ties into morphological development. Although numerous genetics studies have shown that loss or aberrant expression of histone modifying enzymes greatly alters BGC expression and fungal development, no unifying explanation has connected these studies. Here we identify the first chromatin regulatory complex that mechanistically connects tissue development and chemical synthesis. Filamentous fungi differentiate, in increasing complexity, first as hyphae that can be followed by asexual structure or sexual fruiting body formation. Whereas all four KERS mutants still produced hyphae, they are all compromised in asexual spore production, with one of the subunit heterodimers (SntB-RpdA) positively regulating sexual development and the other heterodimer (KdmB-EcoA) having an opposite negative role on sex (Figure 4E–G).

As several of the KERS-targeted genes are known to orchestrate morphological differentiation with SM in filamentous fungi, (e.g. *veA*, *mpkB*, *imeB*), it was expected that SM BGCs are also regulated by KERS. This was exemplified by the impact of KERS mutants on ST BGC expression. ST is a toxic and carcinogenic metabolite whose regulation is studied to understand synthesis of the carcinogen aflatoxin in the related species *Aspergillus flavus* (74). Previous studies have shown that SntB, KdmB and RpdA mutants were all reduced in ST synthesis (18,19,68). We found ST production was coordinated with development by the KERS complex since the ST BGC expression was downregulated in all mutants of the KERS complex (Figure 4J). The influence of KERS on development and ST production is likely to be both direct and indirect since members of the KERS complex bind to both structural and regulatory genes for fungal development and secondary metabolite production (Figure 4). Consequently, inactivation of any of the KERS members leads to a significant loss in ST BGC expression.

A part of KERS complex function seems to be regulated also at the level of protein stability (Figure 5A). EcoA stability is controlled by dual action of SntB and KdmB. KdmB-EcoA interaction protects EcoA from degradation mediated by SntB (Figure 2D). KdmB binding on EcoA presumably masks a degron domain targeted by an unknown kinase cascade and/or ubiquitination mediated by SntB. Although SntB contains PHD domains, its yeast counterpart Snt2 is a bona fide E3 ligase which is responsible for histone degradation (44). Although SntB did not interact stably with EcoA in our purifications, it may still interact with EcoA in a transient manner since E3 ligases often transiently interact with their substrates as described elsewhere (75). In baker's yeast, Eco1 is degraded by the ubiquitin ligase SCF^Cdc4^ not by Snt2 (43). However, in *A. nidulans* EcoA degradation is promoted by SntB. Furthermore, KERS-like complex, Ecm5-Snt2-Rpd3 has not been shown to be associated with Eco1 in yeast (76). Recently, human ESCO2 stability was shown to be controlled by E3 ubiquitin ligase CUL4-DDB1-VPRBP and MCM complexes (77). MCM-ESCO2 physical interaction prevents ESCO2 from proteasomal degradation during cell cycle while CUL4-DDB1-VPRBP promotes ESCO2 degradation during late S phase to suppress cohesion formation. Prevention of ESCO2 degradation by forming a heterodimer with MCM is similar to prevention of EcoA degradation via formation of EcoA-KdmB heterodimer. Presumably both organisms have adopted to stick EcoA homologs to different proteins to protect them from degradation.

Recruitment of EcoA homologs to chromatin or DNA has not been well established. Two recent studies suggest that replicative helicases act as a docking station of ESCO2 and that cohesins recruit ESCO1 in human cells either to mediate centromeric sister chromatid cohesion or to repress transcription (78,79). In our study, in addition to regulation of EcoA stability, KdmB and SntB also recruit EcoA to the promoters at fungal chromatin. This is potentially due to their histone binding properties. However, the function of EcoA at these sites remains unclear. One potential scenario is that EcoA recruitment could be associated with enzymatic properties of KdmB and RpdA which remove H3K4me and H3Ac modifications associated with active transcription. This suggests a possible repressive role for EcoA similar to its human counterparts ESCO2 and ESCO1. While it remains to be determined whether the KERS complex controls cohesin function in *A. nidulans*, our results, nevertheless, suggest a unique mechanism for regulating EcoA function at protein level and chromatin recruitment level.

Presence of histone demethylases and deacetylases in the same complex can be found in mammals and other eukaryotes. There is growing evidence that both enzymes control each other for their catalytic activities positively or negatively when present in the same complex. For example, interaction between both enzymatic activities in LSD1-HDAC complexes has been described. In this case, LSD1 demethylase activity stimulates deacetylation of nucleosomes by HDAC enzymes (80). Consistently, LSD1-Co-REST catalytic activity is decreased with hyperacetylated nucleosomes as substrates suggesting mutual requirement of demethylases with deacetylases (80,81). On the other hand, in *S. cerevisiae* loss of recruitment of Rpd3 to certain genomic loci by *SNT2* or *ECM5* deletion did not result in altered acetylation levels at these sites. This indicates inactivity of Rpd3 when associated with Ecm5, possibly due to inhibition of Rpd3 activity by this demethylase (76). Presence of KdmB and RpdA in the KERS core complex also could indicate a mutual interdependence of their catalytic activities. Interestingly, in our study we do not see an obvious correlation between enzymatic activities (presence of certain modifications) of KdmB and RpdA at recruitment sites (Figure 3E). Of note, histone acetylation is high at the majority of KERS binding sites, which indicates inactivity of RpdA. Furthermore, loss of KdmB led to an increase of HDAC activity of RpdA-SntB dimers *in vitro* (Figure 2K) and *in vivo* (Supplementary Figure S9). Taken together, these facts argue for an inhibition of RpdA HDAC activity by KdmB (or EcoA) when present in the KERS complex. This is similar to observations in Drosophila where interaction of Rdp3 with the LID demethylase complex leads to reduced HDAC activity (82).

KERS is mainly recruited to the locations where the transcription initiation complex subunits such as TBP and TFIID are also recruited (Figure 3F). This is further supported by the positive correlation between KERS bindings and Pol II occupancy in target promoters (Figure 3H). KdmB homologs are recruited to the target promoters by either DNA binding mediator proteins or via their ARID domain which can potentially interact with DNA (83). However, the fact that no discernible transcription factors copurified with KdmB or SntB in our pull down experiments, favours a direct binding to DNA of these proteins. Alternatively, the recruitment may be mediated through interaction(s) with the pre-initiation complex, possibly via TBP that was also pulled down by KdmB.

In summary, the KERS complex is the first eukaryotic chromatin regulatory complex that coordinates development with mycotoxin production in fungi. Its individual members have chromatin binding domains (e.g. PHD and ARID domains in KdmB and SntB) that are thought to mediate the positioning of the complex at their targets. Subsequent histone modifications can be carried out by the intrinsic enzymatic functions such as deacetylation by RpdA or H3K4 de-methylation by KdmB. The components of the KERS complex have been conserved among the eukaryotes and it will be interesting to examine if KERS or KERS-like complexes are conserved both structurally and functionally in other filamentous fungi and perhaps even higher eukaryotes including humans.

## DATA AVAILABILITY

ChIPseq data have been deposited to NCBI SRA database (accession number: PRJNA591094 and PRJNA865103). Genomics data processing and analysis scripts are available on GitHub repository https://github.com/lakhanp1/KERS_project. In addition to excel data sheets, mass spectrometry files of proteomics work are also available from the ProteomeXchange database (accession number: PXD022184). All DNA constructs and strains are available upon request. All other data needed to evaluate the paper are present in the paper or the supplementary material.

## Supplementary Material

gkac744_Supplemental_Files
