# Supplementary material for: The KdmB-EcoA-RpdA-SntB chromatin complex binds regulatory genes and coordinates fungal development with mycotoxin synthesis
Source: Nucleic Acids Res. 2022 Sep 12;50(17):9797–813. doi: 10.1093/nar/gkac744 (PMC9508808; doi:10.1093/nar/gkac744)
Supplement: gkac744_Supplemental_Files [file gkac744_supplemental_files.zip › Karahoda et al 2022 Supplemental Material.pdf]

SUPPLEMENTAL MATERIAL TO

The KdmB-EcoA-RpdA-SntB chromatin complex binds regulatory genes and coordinates fungal development with mycotoxin synthesis

Betim Karahoda[1#], Lakhansing Pardeshi[2,3#], Mevlüt Ulas[1,2#], Zhiqiang Dong[2#], Niranjan Shirgaonkar[2,3], Shuhui Guo[2], Fang Wang[2], Kaeling Tan[2,3], Özlem Sarikaya-Bayram[1], Ingo Bauer[4], Paul Dowling[1], Alastair B. Fleming[5], Brandon T. Pfannenstiel[6], Dianiris Luciano-Rosario[6], Harald Berger[7], Stefan Graessle[4], Mohamed M. Alhussain[5], Joseph Strauss[7], Nancy P. Keller[6], Koon Ho Wong[2,8*], Özgür Bayram[1*]

This file contains following materials:

Supplemental methods

Figure S1. Functionality of KERS complex components fused to TAP, GFP and HA.

Figure S2. Expressions of KERS^{GFP} and ^{TAP} fusion strains along with transcription of EcoA^{HA} fusion in various backgrounds.

Figure S3. ClustalW alignment of EcoA homologs from yeast and human.

Figure S4. Growth of mutant EcoA^{HA} fusion expressing strains in *kdmB*Δ and WT background.

Figure S5. Chromatin association profiles of the KERS subunits for their common bound targets (n = 1,608).

Figure S6. Chromatin association profiles of the KERS subunits in comparison to non KERS target genes.

Figure S7. Expression of KERS complex members and regulatory genes controlled by the KERS complex.

Figure S8. Binding of the KERS complex components on promoters of major asexual and sexual genes.

Figure S9. ChIPseq signal of histone H3 acetylation modifications at promoters bound by the full KERS complex and the RpdA and SntB (RS) subunits.

Supplemental Table S1. DNA Oligonucleotides used in this study.

Supplemental Table S2. Plasmids employed in this study.

Supplemental Table S3. Strains employed in this study.

Supplemental Table S4. A summary of the MEME-ChIP analysis on the common binding sites of the KERS subunits KdmB, EcoA, RpdA and SntB.

Supplemental data S1 to S23 legends

Supplemental methods

Generation and confirmation of *kdmB*, *sntB* deletion and complementation strains

pUC19 (Sigma, Cat# D3404) was used as a recipient plasmid to generate plasmid constructs for *A. nidulans* transformation. *Sma*I site was digested and used to integrate various DNA fragments. *A. nidulans* A4 (FGSC stock centre) genomic DNA was used as template for the amplification of PCR fragments. All DNA fragments were amplified using Q5 High Fidelity DNA Polymerase kit (NEB, Cat# M0491L). *Taq* polymerase was used to verify positive colonies after each bacterial transformation.

For deletion cassettes of *kdmB* and *sntB*, a ~2.0 kbp *ptrA* fragment was released from pME3024 circular plasmid by *Sfi*I digestion. Approximately 1.2 kbp of upstream and terminator regions (OZG610/611, 612/613 for *kdmB*, OZG752/753 and 754/755 for *sntB*) were amplified with primers containing 16 bp overlapping homologous regions with pUC19 and *ptrA* sequence prior to fusing to *ptrA* cassette. Three fragments were then fused into pUC19-*Sma*I region by In-Fusion HD cloning kit (Clontech, Cat# 639650) as described in the product user manual. Resulting plasmids were digested by *Swa*I and transformed into recipient AGB551 strain to create ANOB226 (*kdmB*Δ*::ptrA*) and ANOB254 (*sntB*Δ*::ptrA*). Similarly, *kdmB* and *sntB* mutants were constructed with *pyrG* or *pyroA* markers. *pyrG* cassette was amplified using OZG695/OZG694 oligonucleotides yielding ~1.9 kbp fragment. *pyroA* marker was amplified using OZG696/OZG694 oligonucleotides yielding ~1.6 kbp fragment. These were fused to *kdmB* and *sntB* flanking regions yielding following deletion plasmids pBK53 (*kdmB*Δ*::AfpyrG*) and pBK128 (*sntB*Δ*::pyroA*), respectively. *kdmB*Δ*::AfpyrG* was amplified (BK337/BK338, yielding ~4.2 kbp) from pBK53 and transformed into AGB551 to create ANBK53. Similarly, *sntB*Δ*::pyroA* was amplified (BK568/BK569, yielding ~3.9 kbp) from pBK128 and transformed into ANBK83.1 to create ANBK112.

To generate complementation strain for ANOB226 (*kdmB*Δ*::ptrA*), ~8.4 kbp genomic locus of *kdmB* (BK389/390) was fused into pOSB114 *Swa*I-digested linear plasmid by In-Fusion HD cloning kit yielding pBK74. Similarly, to generate complementation strain for ANOB254 (*sntB*Δ*::ptrA*), ~8.4 kbp genomic locus of *sntB* (BK391/396) was fused into *Swa*I-digested pOSB114 by In-Fusion HD cloning kit yielding pBK75. The resulting complementation plasmids (pBK74, pBK75) were transformed into *kdmB*Δ*::ptrA and sntB*Δ*::ptrA* recipients respectively. *pyroA*$^{+}$ transformants were selected and the mRNA expression levels of *kdmB* and *sntB* were confirmed by RT-qPCR using gene-specific oligonucleotides.

Generation and confirmation of promoter replacement strains for *ecoA* and *rpdA*

In order to generate pOB549, pCH008 was digested by *Pst*I and *Acc65*I to remove *ptrA* marker. OZG1077/11845 was used to amplify *pyroA* cassette (~1.7 kbp) by using pOB508 as a plasmid template. The resulting *pyroA* fragment was fused to *Pst*I/*Acc65*I-digested pCH008 to create pOB549. For generation of *ecoA* (pBK32), *rpdA* (pBK33) Tet-ON-*pyroA* constructs, pOB549 was digested with *Swa*I yielding two fragments for the insertion of upstream and ORF (open reading frame) fragments of *ecoA* and *rpdA*. To generate pBK32, BK127/BK129 were used to amplify ~1.21 kbp fragment of *ecoA* 5'UTR harbouring 16 bp pUC19 overlapping region from 5' end and 16 bp *pyroA* region from 3' end. Similarly, BK130/BK131 were used to amplify ~1.3 kbp ORF region of *ecoA* harbouring 16 bp homology with Tet-ON sequence at 5' end and 16 bp *sgfp* homology at 3' end. Four linear fragments were mixed and fused into circular plasmid by In-Fusion HD cloning kit. The resulting plasmid resembled *tetO7::Pmin::ecoA::pyroA* cassette when amplified by BK128/BK132 yielding ~6.23 kbp of linear fragment. Similarly, for pBK33 construct, BK133/BK135 were used to amplify ~1.22 kbp fragment of *rpdA* 5'UTR harbouring 16 bp

pUC19 overhang region from 5' end and 16 bp *pyroA* region from 3' end. Similarly, BK136/BK137 oligonucleotides were used to amplify ~1.52 kbp ORF region of *rpdA* harbouring 16 bp homology with Tet-ON sequence at 5' end and 16 bp *sgfp* homology at 3' end. Linear fragments harbouring 16 bp homology sequences at 5' and 3' ends were fused to circular plasmid by in-Fusion HD cloning kit. The resulting plasmid resembled *tetO7::Pmin::rpdA::pyroA* cassette when amplified by BK134/BK138 oligonucleotides yielding ~6.56 kbp of linear fragment which was transferred into fungal recipient. Promoter replacements were confirmed by Southern hybridization.

Generation and confirmation of epitope tagged strains; HA, TAP, GFP

For the generation of *ctap*, *sgfp* and *3xha* tagged *kdmB*, *ecoA*, *rpdA*, *sntB* strains, a similar strategy was used to create circular plasmid DNA. These epitope tags were fused into upstream and terminator regions of corresponding genes. *ctap::natR* and *sgfp::nat*R fragments were amplified using OZG916/OZG927 yielding ~1.9 kbp and 2.1 kbp fragments respectively. *3xha::pyrG* fragment was amplified using OZG916/OZG694 oligonucleotides yielding ~2.7 kbp fragment.

In order to create GFP and TAP fusions of KdmB, ORF (open reading frame) of *kdmB* (OZG552/550) and 3'UTR (OZG551/553) were amplified and fused to *ctap::natR* and *sgfp::natR* using fusion PCR (nested oligos OZG548/549), these *kdmB::ctap::natR* and *kdmB::sgfp::natR* PCR products were transformed into WT recipient strain. To construct *sntB::ctap* and *sntB::sgfp* fusions, 1 kb promoter including ORF of *sntB* (OZG1037/1039) and 3'UTR (OZG1040/1041) were amplified and fused to *sgfp* and *ctap* in *Sma*I site of pUC19 leading to plasmids pOB487 (*sntB::sgfp::natR*) and pOB488 (*sntB::ctap::natR*). Cassettes were released by digesting the plasmids with *Pme*I. Similarly, *rpdA* (OZG1033/1034, promoter and ORF, OZG1035/1036 3'UTR) and *ecoA* (OZG1042/1043, promoter and ORF, OZG1044/1045 3'UTR) were amplified and fused to *sgfp* and *ctap*

creating the plasmids, pOB485 (*rpdA::sgfp::natR*), pOB486 (*rpdA::ctap::natR*), pOB489 (*ecoA::sgfp::natR*), pOB490 (*ecoA::ctap::natR*).

To create *kdmB::3xha::AfpyrG* fusion plasmid, *kdmB* ORF was amplified using BK27/BK28 oligonucleotides which overhangs complementary to pUC19 from 5' end and GGGSGG linker from 3'end. 3'UTR region of *kdmB* was amplified using BK29/BK30 where BK29 overhangs with *pyrG* from 5' site and BK30 overhangs pUC19 from 3' site. Three fragments were fused into the *Sma*I-site of pUC19 by in-Fusion HD cloning kit. This resulted in plasmid pBK11 which comprises the *kdmB::3xha::AfpyrG* fusion cassette when amplified by OZG549/OZG552 oligonucleotides yielding ~5.0 kbp fragment.

To create *ecoA*::*3xha*::*AfpyrG* fusion cassette, OZG1042/BK9 were used to amplify *ecoA* fragment from 5'UTR yielding ~1.77 kbp. *Pme*I site and pUC19 15 bp overhang region were introduced to 5' end of OZG1042. BK8/OZG1045 were used to amplify ~2.2 kbp region of *ecoA* 3'UTR. Similarly, 15 bp overhang sequence of *pyrG* 3'end was introduced into 5' end of BK8. *Pme*I and 15 bp overhang sequence of pUC19 was introduced into 5'end of OZG1045. Three fragments were fused into the *Sma*I-site of pUC19 resulting in plasmid pBK3 which comprises the *ecoA::3xha::AfpyrG* fusion cassette when digested by *Pme*I restriction enzyme yielding ~6.6 kbp linear fragment.

To create *rpdA*::*3xha*::*AfpyrG* fusion cassette, OZG1033/BK2 were used to amplify *rpdA* fragment from 5'UTR yielding ~2.7 kbp. *Pme*I site and pUC19 15 bp overhang region were introduced to 5' end of OZG1033. BK5/OZG1036 were used to amplify 806 bp region of *rpdA* 3'UTR. Similarly, 15 bp overhang sequence of *pyrG* 3'end was introduced into 5' end of BK5. *Pme*I and 15 bp overhang sequence of pUC19 was introduced into 5'end of OZG1036. Three fragments were fused into the *Sma*I-site of pUC19 resulting in plasmid pBK1 which comprises the *rpdA::3xha::AfpyrG* fusion cassette when digested by *Pme*I restriction enzyme yielding ~6.2 kbp linear fragment.

To create *sntB::3xha::AfpyrG* fusion cassette, OZG1037/BK7 were used to amplify *sntB* ORF yielding ~5.9 kbp. *Pme*I site and pUC19 15 bp overlapping region were introduced to 5' end of OZG1037. BK6/OZG1041 were used to amplify ~1.23 kbp region of *sntB* 3'UTR. Similarly, 15 bp overlapping sequence of *pyrG* 3'end was introduced into 5' end of BK6. *Pme*I and 15 bp overlapping sequence of pUC19 was introduced into 5'end of OZG1041. Three fragments were fused into the *Sma*I-site of pUC19 resulting in plasmid pBK2 which comprises the *sntB*::*3xha*::*pyrG* fusion cassette when digested by *Pme*I restriction enzyme yielding ~9.6 kbp linear fragment.

To create *sudA::3xha::AfpyrG* fusion cassette, *3xha*::*AfpyrG* fragment was amplified using OZG916/694 from plasmids pOB430. To create pBK87, ORF (BK444/BK445) and 3' UTR (BK435/BK436) regions were amplified by using the WT genomic DNA of A4 strain. These two fragments and *3xha*::*AfpyrG* were fused to *Sma*I-digested pUC19 by using In-Fusion HD Cloning kit, yielding pBK87 from which *sudA*::*3xha*::*AfpyrG* cassette was amplified (BK446/438) and transformed into recipient fungal strains AGB551 (WT), *tetO7::Pmin::ecoA::pyroA* (ANBK33.1) and ANOB226 (*kdmB*Δ*::ptrA*). *pyrG*+ transformants were selected in the absence of uridine, uracil and epitope tags of *sudA* were confirmed by Southern hybridization.

Site directed mutagenesis of *ecoA* with 3XHA fusion

In order to generate mutated versions of *ecoA* following general strategy was performed. Two fragments were amplified from pBK3 *ecoA::3xha::AfpyrG* plasmid. (I) Promoter region and N-terminal sequence (until S41 and S45) of *ecoA* (700 bp) was amplified with a primer pair. (II) From S41 and S45 until Terminator sequence (3 UTR) (6 Kbp) was amplified with another primer pair. These two PCR fragments contained 15 bp identical regions which were used to clone these two fragments into *Sma*I site of pUC19 using HD Cloning kit. Positive

clones detected in colony PCR were grown for plasmid preparation which was followed by sequencing in GATC (Germany). For pOB559 (S41>A41): Fragment I (OZG1042/1258), Fragment II (OZG1259/1045) were amplified from pBK3 and fused in *Sma*I site of pUC19. For pOB560 (S45>A45) : Fragment I (OZG1042/1256) and Fragment II (OZG1257/1045) were fused in *Sma*I site of pUC19. For pOB561 (S41-S45>A41-S45) : Fragment I (OZG1042/1237) and Fragment II (OZG1238/1045) were used. For pOB562 (S41>D41) : Fragment I (OZG1042/1239) and Fragment II (OZG1240/1045) were used. For pOB563 (S45>D45) : Fragment I (OZG1042/1241) and Fragment II (OZG1242/1045) were used. For pOB564 (S41-S45>D41-D45) : Fragment I (OZG1042/1243) and Fragment II (OZG1244/1045) were used. Once confirmed with SANGER sequencing (Eurofins), these gene replacement fragments were released from these plasmids using *Pme*I digestion, which released the 6.5 kbp gene replacement cassettes. These cassettes were eventually transformed into AGB551 and *kdmB*Δ*::ptrA*. Gene replacements were confirmed by Southern hybridization. Following strains were generated from AGB551: ANOB559 (S41>A41), ANOB560 (S45>A45), ANOB561(S41-S45>A41-S45), ANOB562 (S41>D41), ANOB563 (S45>D45), ANOB564 (S41-S45>D41-D45). Following strains were generated from ANBK83.1: ANOBKD559 (S41>A41), ANOBKD560 (S45>A45), ANOBKD561(S41-S45>A41-S45), ANOBKD562 (S41>D41), ANOBKD563 (S45>D45), ANOBKD564 (S41-S45>D41-D45).

Generation of deletion constructs for *sem1*

In order to generate pOB566 (*sem1*Δ*::pyroA*) containing proteasome subunit encoding gene *sem1,* pME4567 plasmid, a gift of Prof. Gerhard Braus was digested with *EcoR*I/*Nde*I to remove *ptrA* cassette and PCR-amplified *pyroA* marker (cassette) (OZG1251/1252) was cloned in pME4567 using HD cloning. A 5.8 kbp *sem1*Δ*::pyroA* cassette was released by

digesting with *Hind*III, which was ultimately transformed into ANBK83.1 (*kdmB*Δ*::ptrA*, *ecoA::3xha*) leading to strain ANBK83.1.566. Phenotype was confirmed with growth test which was characterised by drastically reduced colony size as published previously (1) (Figure S4).

Generation of BIFC plasmids for *in vivo* protein-protein interaction

For *in vivo* interaction analyses, *n-eyfp* (OZG73/74) and *kdmB* cDNA (OZG670/OZG671) were amplified and cloned into *Swa*I site of pSK353 generating pOB282 plasmid. Similarly, *c-eyfp* (OZG75/76) and *kdmB* cDNA (OZG673/OZG671) were amplified and cloned into *Swa*I site of pSK353 generating pOB283 plasmid. *rpdA* cDNA was amplified (OZG857/OZG859) and fused to *c-eyfp* (OZG677/OZG388) and combined with n-*eyfp kdmB* (inserted into *PmeI*-digested pOB282) generating plasmid pOB302. *rpdA* cDNA was amplified (OZG858/OZG859) and fused to *n-eyfp* (OZG674/OZG387) and combined with c-*eyfp kdmB* (inserted into *Pme*I-digested pOB283) generating plasmid pOB303. The appropriate *n-eyfp*::*kdmB*, *c-eyfp*::*rpdA* or *n-eyfp*::*rpdA*, *c-eyfp*::*kdmB* fusion constructs were transformed into recipient WT fungal strain to detect *in vivo* KdmB and RpdA interaction.

sntB cDNA was amplified (OZG860/OZG862) and fused to *c-eyfp* (OZG677/OZG388) and combined with n-*eyfp kdmB* (inserted into *PmeI*-digested pOB282) generating plasmid pOB304. *sntB* cDNA was amplified (OZG861/OZG862) and fused to *n-eyfp* (OZG674/OZG387) and combined with c-*eyfp kdmB* (inserted into *Pme*I-digested pOB283) generating plasmid pOB305. The appropriate *n-eyfp*::*kdmB*, *c-eyfp*::*sntB* or *n-eyfp*::*sntB*, *c-eyfp*::*kdmB* fusion constructs were transformed into recipient WT fungal strain to detect *in vivo* KdmB and SntB interaction.

ecoA cDNA was amplified (OZG863/OZG865) and fused to *c-eyfp* (OZG677/OZG388) and combined with n-*eyfp kdmB* (inserted into *PmeI*-digested pOB282) generating plasmid

pOB306. *ecoA* cDNA was amplified (OZG864/OZG865) and fused to *n-eyfp* (OZG674/OZG387) and combined with c-*eyfp kdmB* (inserted into *Pme*I-digested pOB283) generating plasmid pOB307. The appropriate *n-eyfp*::*kdmB*, *c-eyfp*::*ecoA* or *n-eyfp*::*ecoA*, *c-eyfp*::*kdmB* fusion constructs were transformed into recipient WT fungal strain to detect *in vivo* KdmB and EcoA interaction.

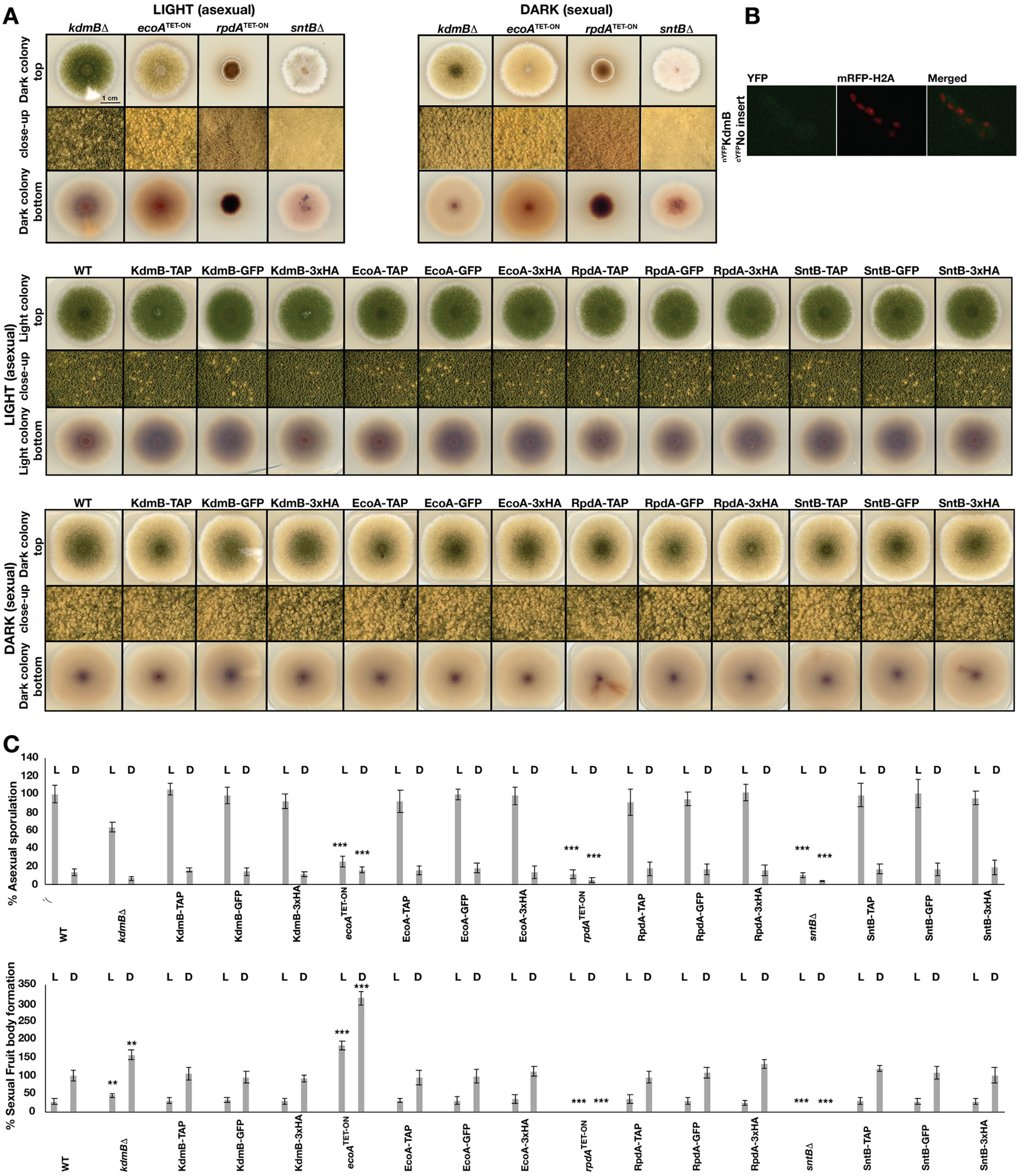

Figure S1. Functionality of KERS complex components fused to TAP, GFP and HA. Growth of WT, KdmB, EcoA, RpdA and SntB-TAP, GFP and HA strains in comparison to *kdmB*Δ, *ecoA*Δ (lethal), *rpdA*Δ (lethal) and *sntB*Δ. **(A)** Upper panel: Asexual growth of the strains ($1x10^3$) on GMM plates at 37 ºC for 5 days under continuous white light. Colony top and bottom pictures along with stereomicroscopic images are shown. Lower panel: Sexual growth of the strains on GMM plates at 37 ºC for 5 days without light. TAP/GFP/HA-tagged

strains behave similar to WT strains for sporulation, growth and development. (**B**) Control for BIFC experiments. ^{nYFP}KdmB is expressed with cYFP within the same fungal strain. mRFP-H2A indicates the position of nuclei. Cells were grown under same conditions as in Figure 1D. **(C)** Quantification of asexual and sexual structures from (A). WT asexual sporulation and sexual development set as 100% capacity. Asexual sporulation and sexual development of the mutants and tagged strains in comparison to WT strain. All fusion strains produce similar number of asexual and sexual structures of the WT strain.

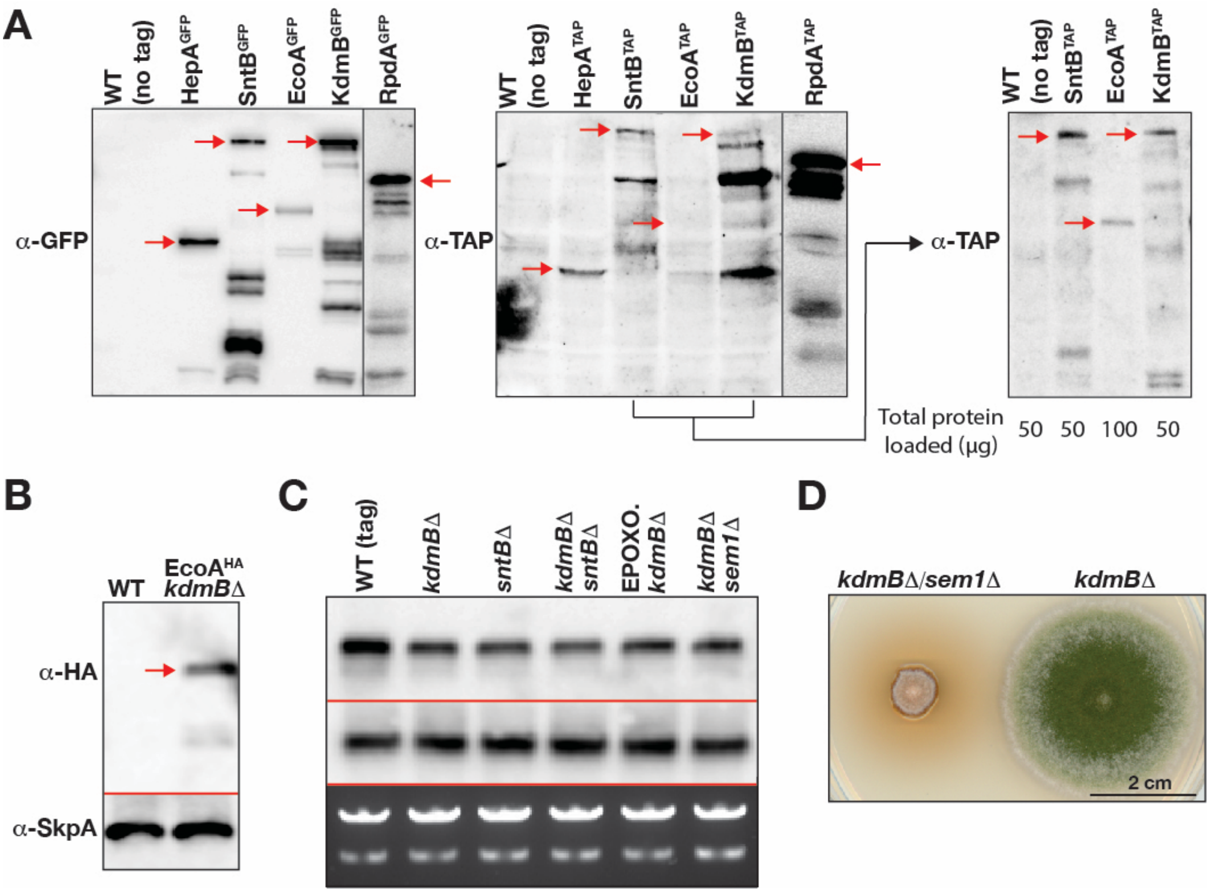

Figure S2. Expressions of KERS^{GFP} and ^{TAP} fusion strains along with transcription of EcoA^{HA} fusion in various backgrounds. (**A**) Expression of KERS complex components fused to GFP and TAP epitope tags. α-GFP and α-TAP antibodies were applied on 100 µg protein extracts. RpdA^{GFP} or RpdA^{TAP} fusion were run on different blots (separated with a line) due to their strong signals which prevent detection of KdmB, EcoA, SntB^{GFP} or ^{TAP} fusion signals. For EcoA^{TAP}, a separate Western blot analysis using reduced loading amounts of SntB^{TAP} and KdmB^{TAP} extracts was performed to show EcoA^{TAP} expression. Red arrows indicate approximate MW of the fusion proteins. HepA^{GFP}; 50 kDA, SntB^{GFP}; 200 kDA, EcoA^{GFP}; 70 kDA, KdmB^{GFP}; 220 kDA, RpdA^{GFP}; 105 kDA, HepA^{TAP}; 45 kDA, SntB^{TAP}; 195 kDA, EcoA^{TAP}; 65 kDA, KdmB^{TAP}; 210 kDA, RpdA^{TAP}; 100 kDA. Heterochromatin protein HepA^{GFP} and HepA^{TAP} fusions were used as a positive control. (**B**) Detection of trace amount of EcoA^{HA} fusion in *kdmB*Δ background when compared to WT (no tag) control. (**C**)

Transcriptional expression of *ecoA* fusions in WT, *kdmB*Δ, *sntB*Δ, *kdmB*Δ/*sntB*Δ and *kdmB*Δ/*sem1*Δ double and epoximycin treated cells. Total 20 µg of RNA was loaded. Glycolytic *gpdA* gene and rRNA bands serve as loading control. (**D**) Confirmation of *sem1*Δ phenotype in *kdmB*Δ/*sem1*Δ double mutant in comparison to *kdmB*Δ mutant strain. *kdmB*Δ and *kdmB*Δ/*sem1*Δ double mutant strains were grown on GMM plates at 37 ºC for 3 days under continuous white light.

288

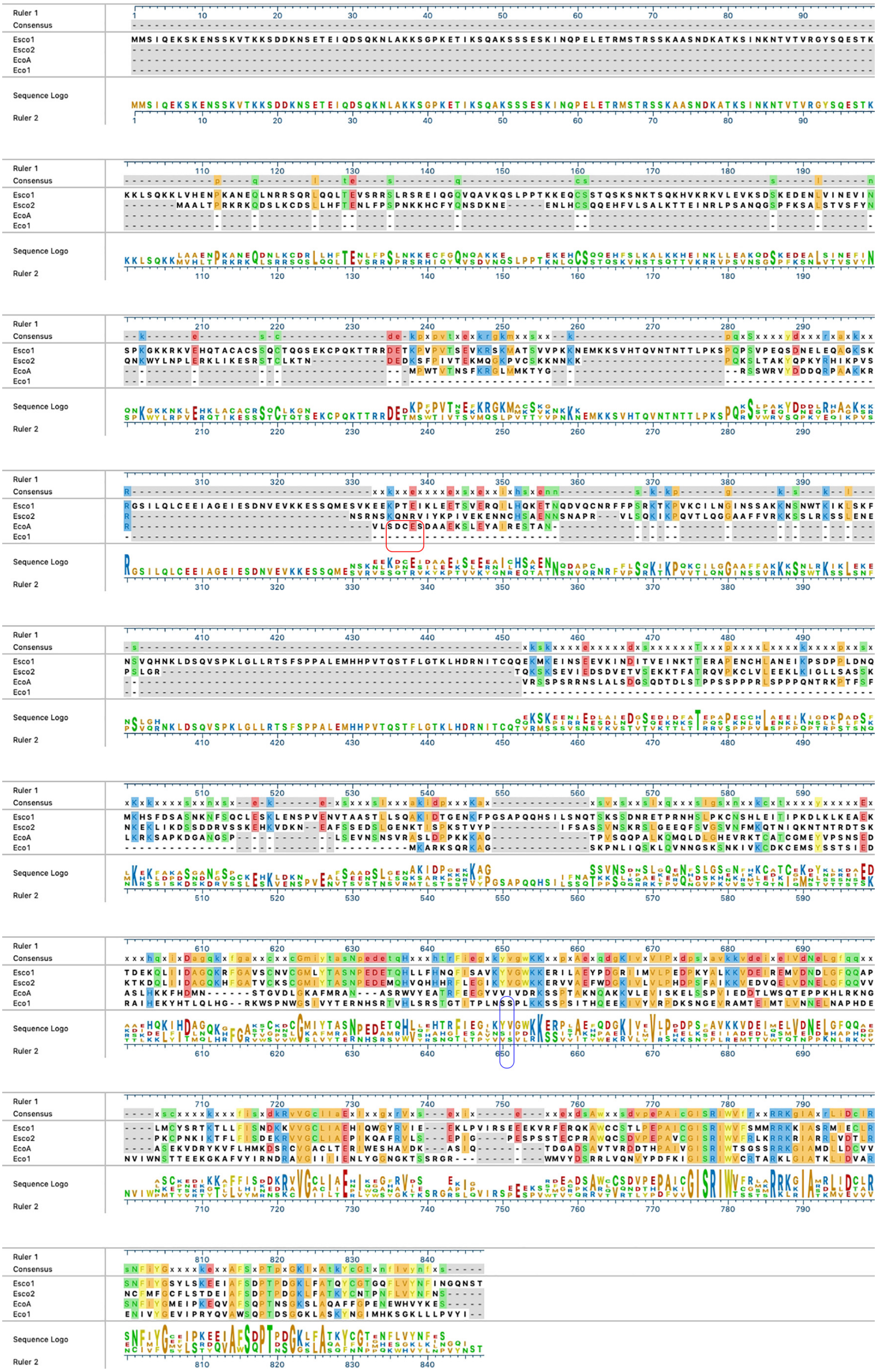
Ruler 1
Consensus
Esco1
Esco2
EcoA
Eco1
Sequence Logo
Ruler 2

Figure S3. Clustal W alignment of EcoA homologs from yeast and human. Human ESCO1 (840 aa) and ESCO2 (601 aa) is longer than both yeast Eco1 and Aspergillus EcoA. All EcoA homologs show a conserved C-terminal sequence. N-terminus of the proteins show diversity. Two Ser residues 41 and 45 in EcoA is indicated with red rectangle. Yeast Eco1 residues involved in stability of Eco1 is shown with blue buble.

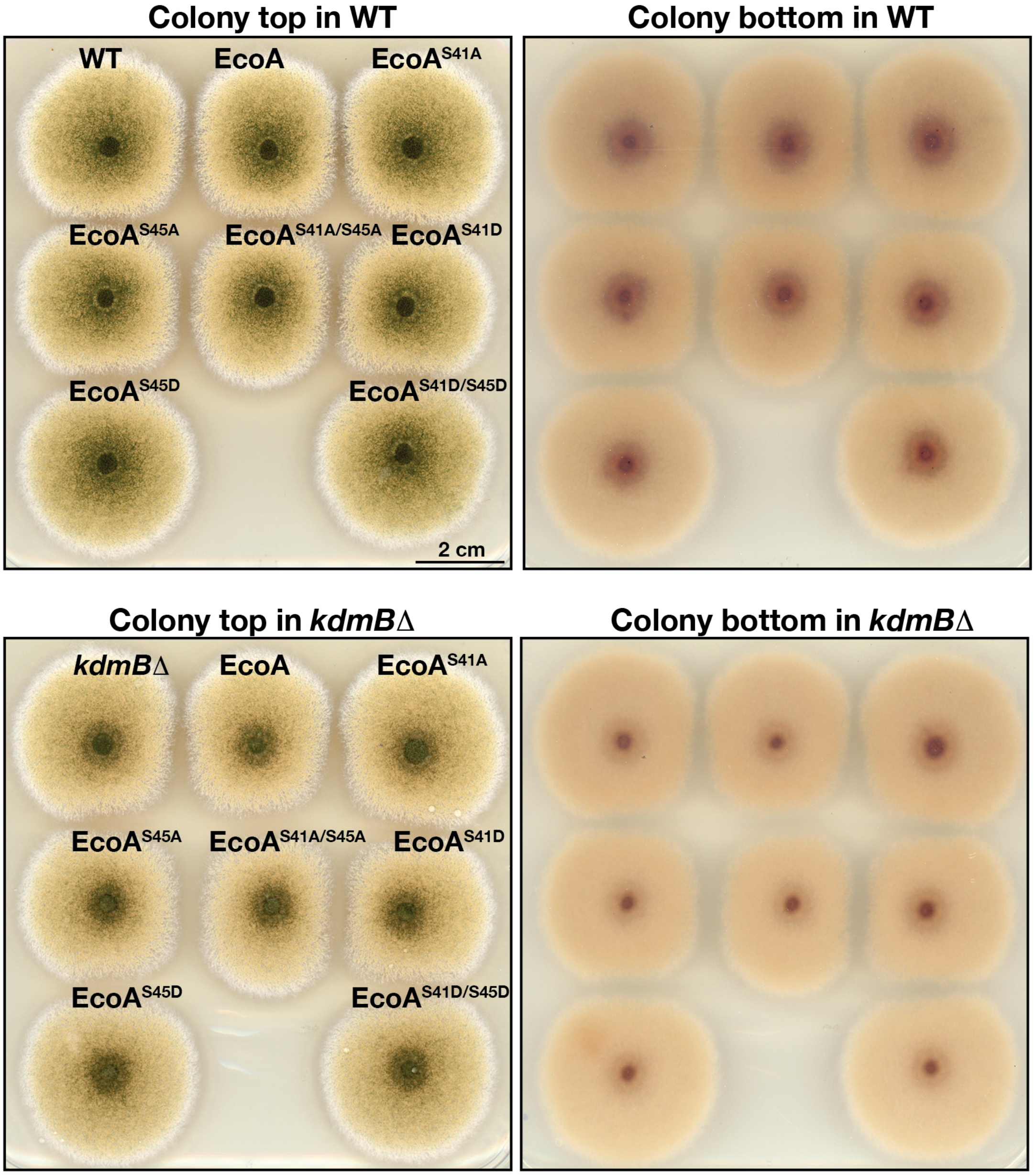

Figure S4. Growth of mutant EcoA^{HA} fusion expressing strains in *kdmB*Δ and WT background. Growth of WT, EcoA^{HA} fusion and mutant (S41A, S45A, S41A-S45A, S41D, S45D, S41D/S45D) versions expressing strains in comparison to *kdmB*Δ. Colony top and bottom pictures along with stereomicroscopic images are shown. Sexual growth of the strains on GMM plates at 37 ºC for 5 days without light.

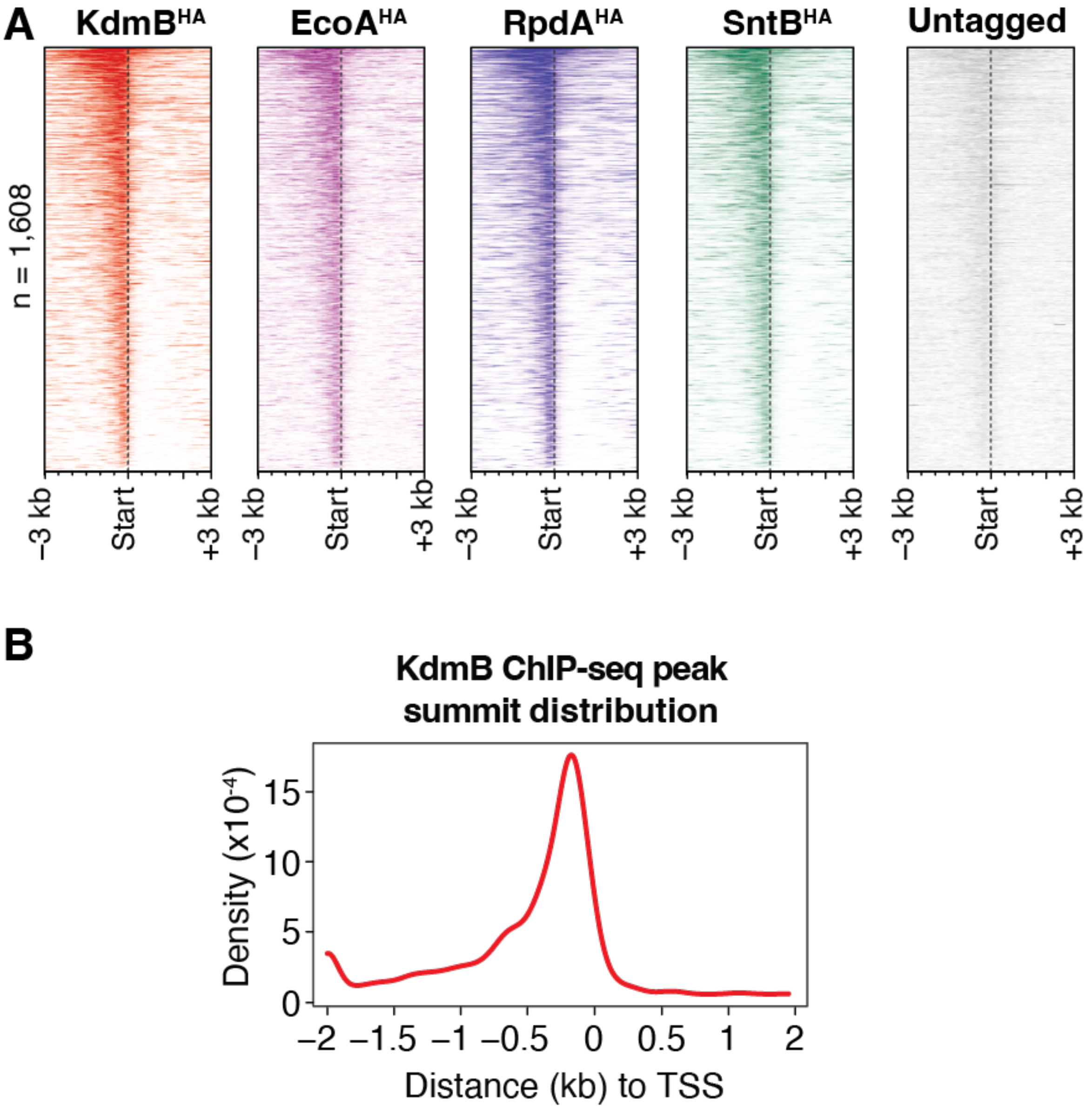

Figure S5. Chromatin association profiles of the KERS subunits. (A) Heatmaps displaying binding locations, signals and intensities of $KdmB^{HA}$, $EcoA^{HA}$, $RpdA^{HA}$ and $SntB^{HA}$ over their target genes. (**B**) Density plot of $KdmB^{HA}$ peak summit distance to the nearest Transcription Start Site (TSS).

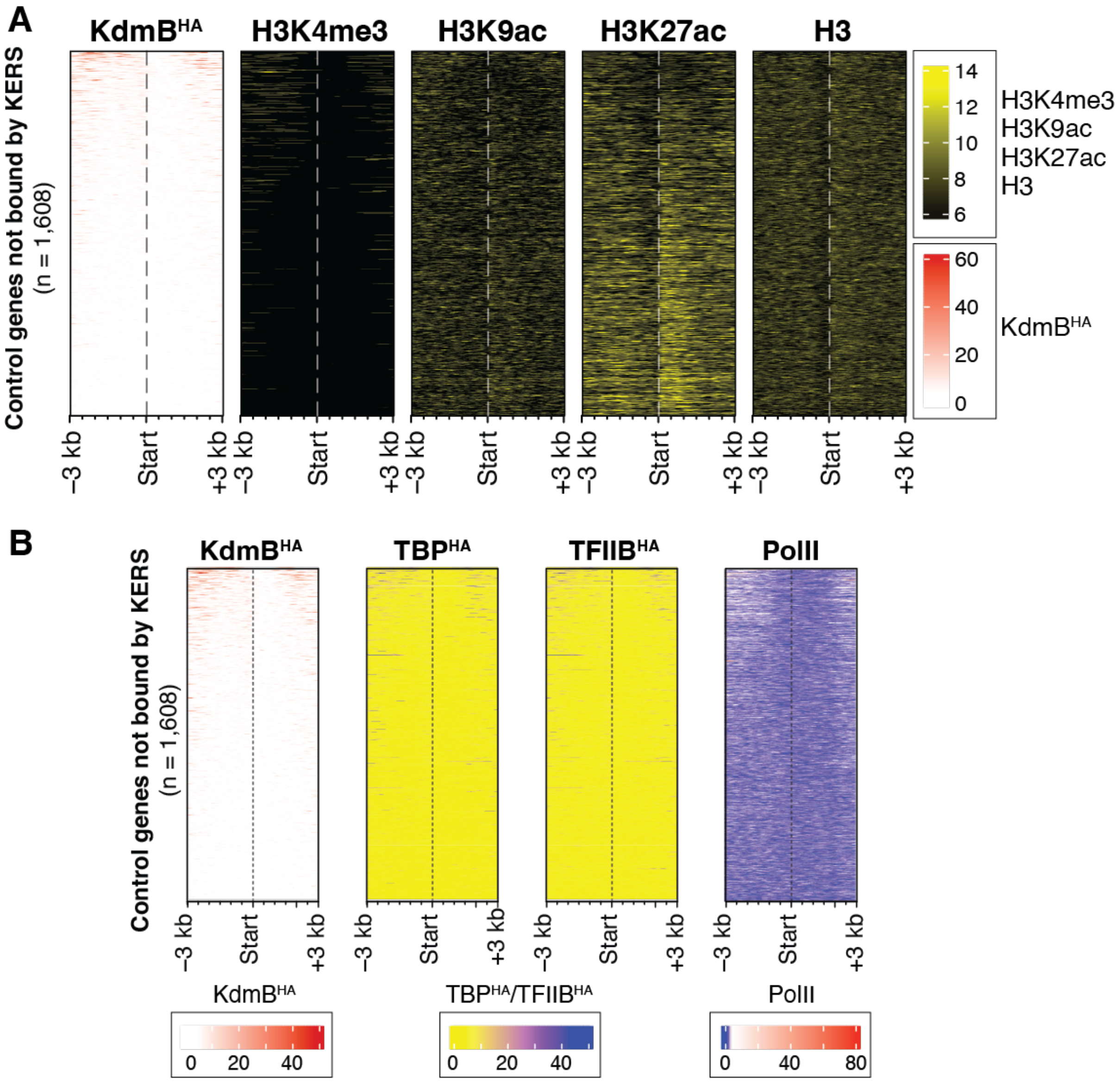

Figure S6. Chromatin association profiles of the KERS subunits in comparison to non KERS target genes. (**A**) Heatmaps displaying ChIPseq signals of KdmB^{HA}, histone H3 lysine 4 trimethylation (H3K4me3), histone H3 lysine 9 acetylation (H3K9ac), histone H3 lysine 27 acetylation, histone H3. (**B**) TBP^{HA} and TFIIB^{HA} at the -3 kb to +3 kb region with respect to the start codon (ATG) of non KERS target genes.

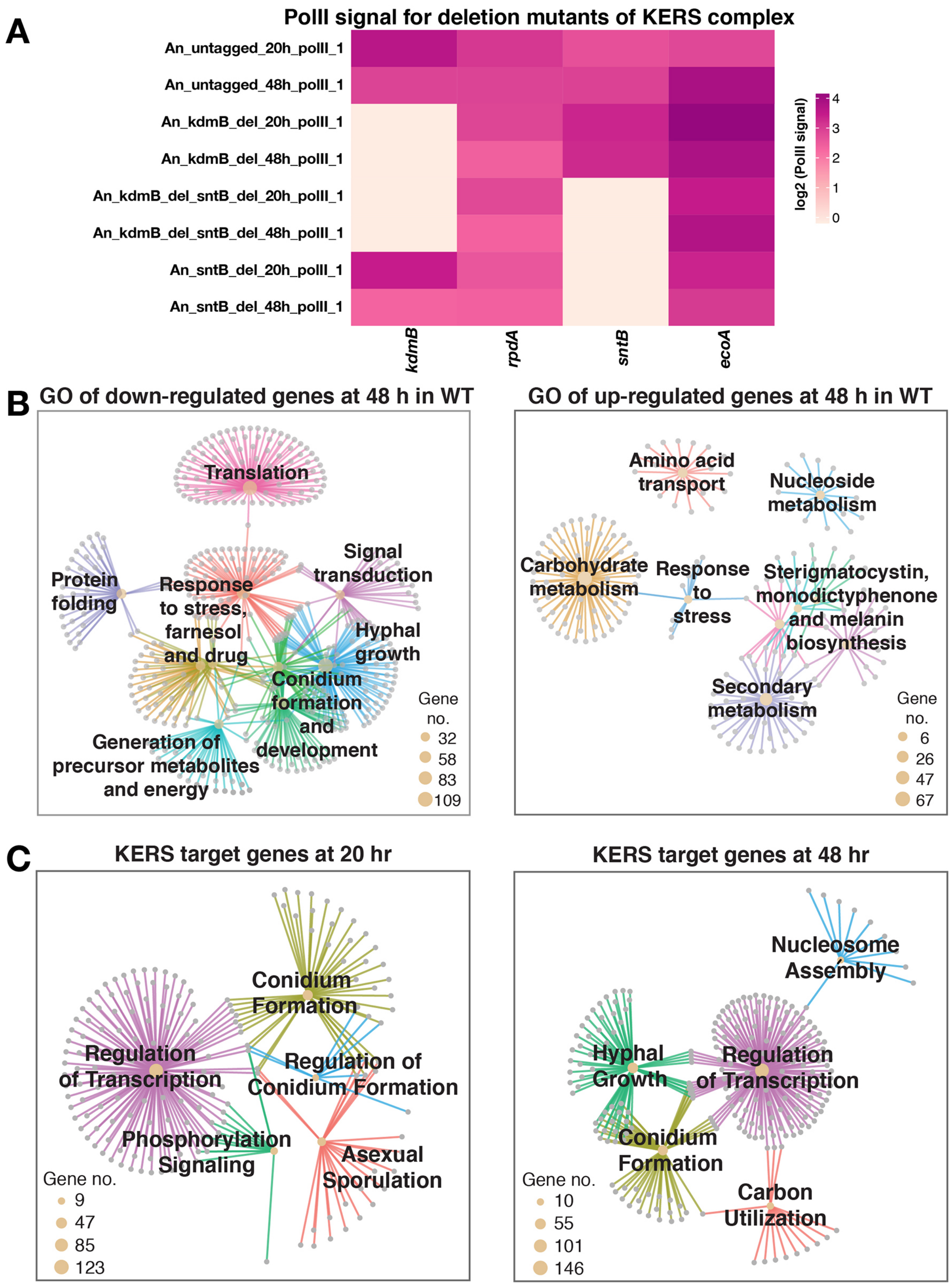

377

378 **Figure S7. Expression of KERS complex members and regulatory genes controlled by**
379 **the KERS complex. (A)** Heat map plot expression of the KERS complex members in the
380 mutants of *kdmB* and *sntB* **(B)** Cytoscape network plots highlighting the top physiological

pathways enriched among genes up- or down-regulated during primary (20 hr) to secondary (48 hr) growth phase transition in WT. **(C)** Cytoscape network plots highlighting the top physiological pathways enriched among genes bound by the full KERS complex during primary and secondary growth phases.

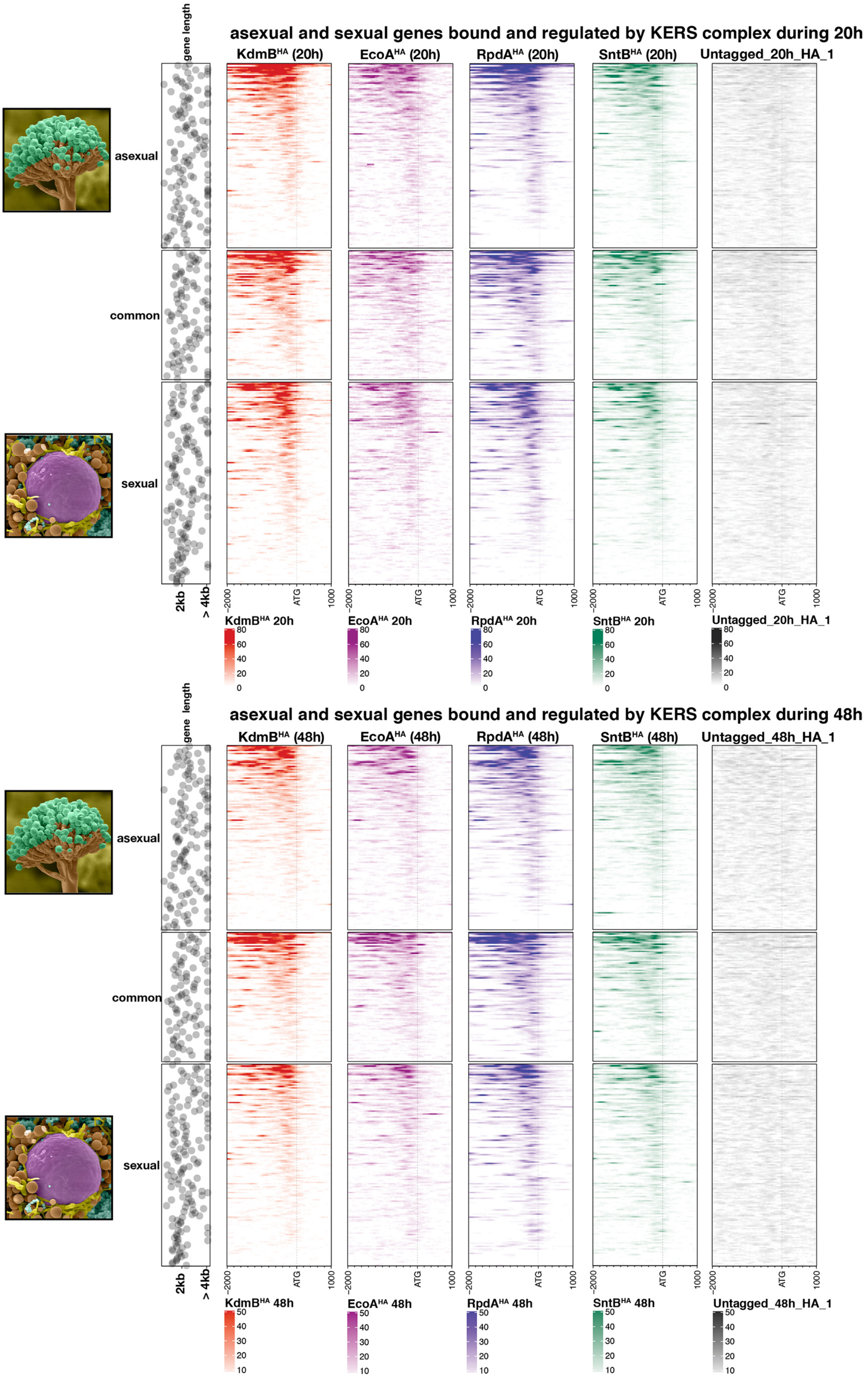
asexual and sexual genes bound and regulated by KERS complex during 20h
gene length
KdmBHA (20h)
EcoAHA (20h)
RpdAHA (20h)
SntBHA (20h)
Untagged_20h_HA_1
asexual
common
sexual
2kb
> 4kb
-2000
ATG
1000
KdmBHA 20h
EcoAHA 20h
RpdAHA 20h
SntBHA 20h
Untagged_20h_HA_1
80
60
40
20
0
asexual and sexual genes bound and regulated by KERS complex during 48h
KdmBHA (48h)
EcoAHA (48h)
RpdAHA (48h)
SntBHA (48h)
Untagged_48h_HA_1
KdmBHA 48h
EcoAHA 48h
RpdAHA 48h
SntBHA 48h
Untagged_48h_HA_1
50
40
30
20
10

Figure S8. Binding of the KERS complex components on promoters of major asexual and sexual genes. Heatmap plots showing $KdmB^{HA}$, $EcoA^{HA}$, $SntB^{HA}$ and $RpdA^{HA}$ ChIPseq signals across upstream 2kb promoter region to 1kb of coding region of genes annotated as sexual or asexual genes or genes implicated in both sexual and asexual development (common).

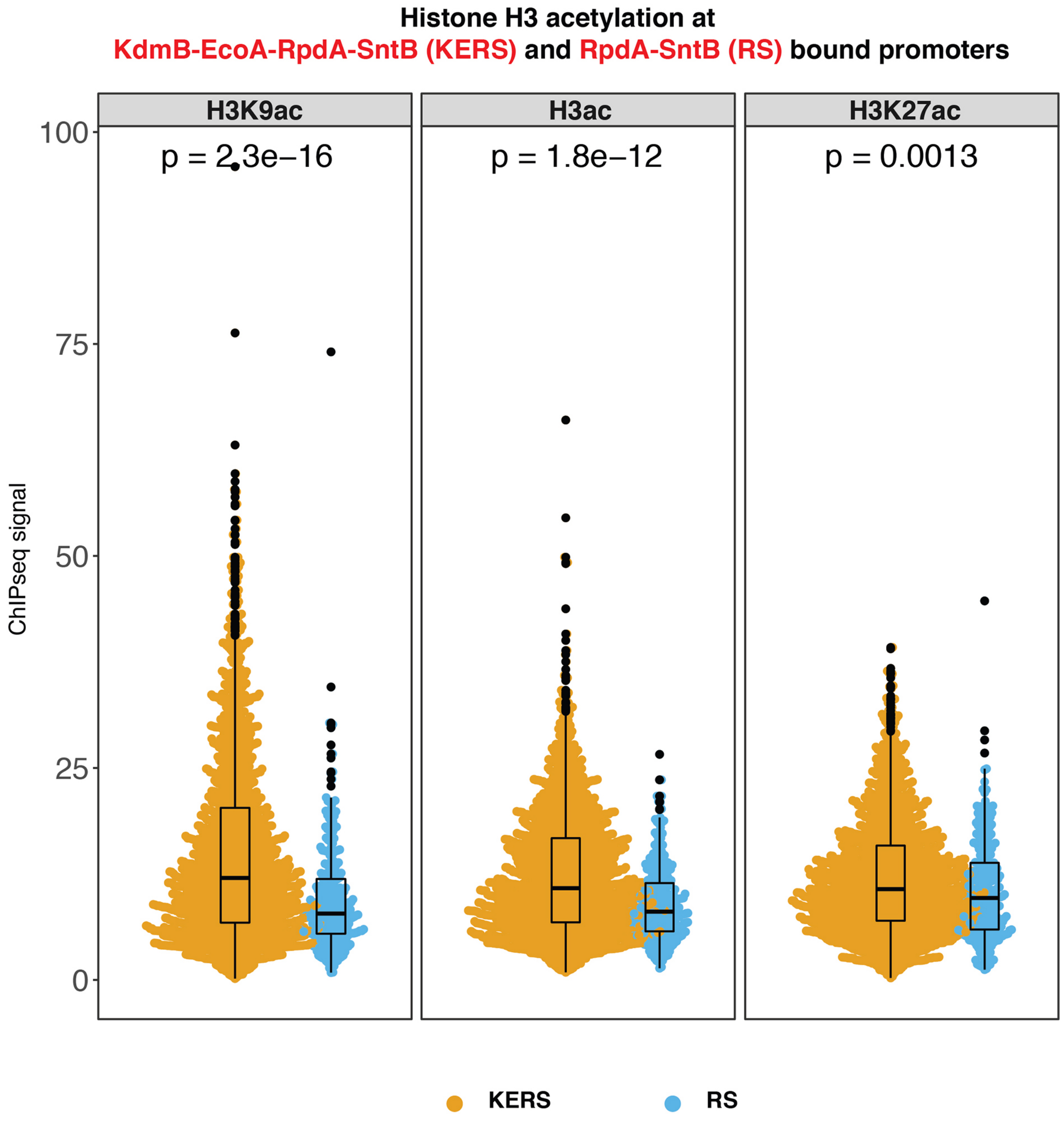

Figure S9. ChIPseq signal of histone H3 acetylation modifications at promoters bound by the full KERS complex and the RpdA and SntB (RS) subunits. Violin plots showing the ChIPseq signals of H3K9ac, H3ac and H3K27ac for the promoters bound by the four KERS subunits and the RpdA and SntB subunits only.

Supplemental Tables

Supplemental Table S1. DNA Oligonucleotides used in this study.

**Designation**	**Sequence in 5' > 3' direction**
BK2 (rpdA RVS)	CACCGCTACCACCTCCCTCCTTTGCGGCTTCGGGTT
BK5 (rpdA 3 UTR FWD)	CCTCCTCTCAGACAGGGACTAGCGCAGCAATTTTGG
BK6 (sntB 3 UTR FWD)	CCTCCTCTCAGACAGCTTGGTCGTAGAGAGATCAAGG
BK7 (sntB RVS)	CACCGCTACCACCTCCGGACAGGAGGTTCTTCAGCG
BK8 (ecoA 3 UTR FWD)	CCTCCTCTCAGACAGTGGTTTGTGCACAATTGTCATTCG
BK9 (ecoA RVS)	CACCGCTACCACCTCCACTCTCCTTGTAGACATGCCA
BK27 (kdmB-*Sma*I-pUC tail FWD PmeI-site)	TCGAGCTCGGTACCCAGCAAACTCATGTTTACAACCAATG
BK28 (kdmB-3xHA tail RVS)	GATAACCACCGCTACCACCTCCCGCAGTGGCTTCGACTTCC
BK29 (kdmB 3 UTR-pyrG tail FWD)	GCCTCCTCTCAGACAGTTCCATCTGCCTCATTCAACATC
BK30 (kdmB-*Sma*I-pUC tail RVS PmeI-site)	ACTCTAGAGGATCCCCGTTGGGATATGCAAGGTGTGC
BK127 (5 UTR ecoA pUC tail)	aaagctgggtacATTTGGTACTTGCATGGATTAGCGATTG
BK128 (5 UTR ecoA nest)	CGCAGTCTTGGACCCATGATG
BK129 (5 UTR ecoA pyrG/pyroA tail)	CCTCTCAGACAGATTTCTCCTTTTTGTACTTCTCCCGAG
BK130 (ORF ecoA tet-ON tail)	gcctgagtggccgtttATGCCGTGGACTGTAACAAATTCAT
BK131 (ORF ecoA GFP tail)	cttgctcaccatgtttCTCTCCTTGTAGACATGCCATTC
BK132 (ORF ecoA nest)	ATGCTTGGGCTAGGCTCTTCC
BK133 (5 UTR rpdA pUC tail)	aaagctgggtacATTTTATCAACGGAGGAGAGCCAGC
BK134 (5 UTR rpdA nest)	TCCAAACCCAGGATGCGAGTG
BK135 (5 UTR	CCTCTCAGACAGATTTTATTTTTGGTAAGTTCGAGGGTAAGG

rpdA pyrG/pyroA tail)	
BK136 (ORF rpdA tet-ON tail)	gcctgagtggccgtttATGGCTTCGGGAACGTCGGG
BK137 (ORF rpdA GFP tail)	cttgctcaccatgtttCGCCGGGCTTCTCAACATATTG
BK138 (ORF rpdA nest)	ATCAAAGCGTCGCTTTGTAAAGCG
BK212 (pyroA control RVS)	CACCACCCTTCAGCATCTGAG
BK213 (pyroA control FWD)	GGTATCAATGTCTCTCAGATGCC
BK214 (kdmB 5 UTR OUT)	GAT GGG GTG AAT GGG TTG AAC
BK215 (ecoA 5 UTR OUT)	CGCTGCATCCAGAATTGAGCG
BK216 (rpdA 5 UTR OUT)	GTCCAGACTTGCTTGTGCAGG
BK217 (sntB 5 UTR OUT)	GCAGCACGCTTCACCAGAAC
BK224 (kdmB Rvs cDNA)	ACTTCCGGCTCAGGTTGAC
BK225 (kdmB Fwd cDNA)	CCTGAACCTATGGAGAATACTC
BK226 (ecoA Fwd cDNA)	CACGCATCTGGACGTCTG
BK227 (ecoA Rvs cDNA)	TGGCTGAAAGCTACCTGTTC
BK228 (rpdA Fwd cDNA)	CAGCAGTGACAACCGAGAC
BK229 (rpdA Rvs cDNA)	GTTGTGCTTCTGCGCTAGG
BK230 (sntB Fwd cDNA)	CTACAGGCAGCACACCAG
BK231 (sntB Rvs cDNA)	GTGTCGAGGTCCCAATCG
BK244 (sconC Fwd cDNA)	CCAACTACCTTGACATCAAGC
BK245 (sconC Rvs cDNA)	GATCTGGTCCTCTTCCTCG
BK280 (benA qPCR F)	GATGGCTGCCTCTGACTTC
BK281 (benA qPCR R)	GCATCTGGTCCTCAACCTC
BK333 (kdmB 5 UTR pUC tail)	TTCGAGCTCGGTACCCCTTGCAAAGATCAGCTCTGATTCAG

BK334 (kdmB 5 UTR pyrG tail)	GAGCATTGTTTGAGGCGTCTTGGGTGCGATAGCTGG
BK335 (kdmB 3 UTR pyrG/pyroA tail)	GCCTCCTCTCAGACAGTTCCATCTGCCTCATTCAACATCT
BK336 (kdmB 3 UTR pUC tail)	ACTCTAGAGGATCCCCGTTGGGATATGCAAGGTGTGCG
BK337 (kdmB 5 UTR nest)	GAGTGGAACCTCTGTTGTGTAC
BK338 (kdmB 3 UTR nest)	AGGAGAAGGACATTCTGGGATC
BK389 (kdmB 5 UTR F pUC tail compl)	AGCTCGGTACCCATTTTGGATTGAACGAGTTACCATCTAC
BK390 (kdmB 3 UTR R pUC tail compl)	CATCTGATGTCCATTTAGTTACAACTCTCGTCACAGAGG
BK390 (kdmB 3 UTR R pUC tail compl)	CATCTGATGTCCATTTAGTTACAACTCTCGTCACAGAGG
BK391 (sntB 5 UTR F pUC tail compl)	AGCTCGGTACCCATTTCACCATCCCACTACGACTGC
BK396 new (sntB 3 UTR R pUC tail compl)	CATCTGATGTCCATTTCCAGGATTGTAGAGGTTTGAGC
BK397 (sntB 5 UTR F)	CCCAGTCGATCACTGGGTTAC
BK398 (sntB 3 UTR R)	TCGTCGTGCTCTCCTTATCATG
BK435 (sudA 3 UTR pyrG tail)	GCCTCCTCTCAGACAGGATGATGGTGATGATCAGTTCCCT
BK436 (sudA 3 UTR pUC tail)	ACTCTAGAGGATCCCCCAGTGATGTTCTCGAGCGATAG
BK438 (sudA 3 UTR nest)	CAGTGATGTTCTCGAGCGATAG
BK444 (sudA 5 UTR pUC tail)	TTCGAGCTCGGTACCCGGTTCCGAGCTGCTGATTGG
BK445 (sudA ORF HA tail)	CACCGCTACCACCTCCTGACTTCTGCTCCTCGACAAATTTG
BK446 (sudA 5 UTR nest)	GGTTCCGAGCTGCTGATTGG
BK564 (5 UTR sntB pUC tail)	TTCGAGCTCGGTACCCCCCAGTCGATCACTGGGTTAC
BK565 (5 UTR sntB pyroA tail)	CCAGCATCTGATGTCCCCTCCGACGCGACAAGAAAAG

BK566 (sntB ORF pyroA tail)	GCCTCCTCTCAGACAGGCTCACAAAGGACACTGTTGCTG
BK567 (sntB ORF pUC tail)	ACTCTAGAGGATCCCCTCGTCGTGCTCTCCTTATCATG
BK568 (5 UTR sntB nest F)	CCCAGTCGATCACTGGGTTAC
BK569 (ORF sntB nest R)	TCGTCGTGCTCTCCTTATCATG
BK564 (5 UTR sntB pUC tail)	TTCGAGCTCGGTACCCCCCAGTCGATCACTGGGTTAC
BK565 (5 UTR sntB pyroA tail)	CCAGCATCTGATGTCCCCTCCGACGCGACAAGAAAAG
BK566 (sntB ORF pyroA tail)	GCCTCCTCTCAGACAGGCTCACAAAGGACACTGTTGCTG
BK567 (sntB ORF pUC tail)	ACTCTAGAGGATCCCCTCGTCGTGCTCTCCTTATCATG
BK568 (5 UTR sntB nest F)	CCCAGTCGATCACTGGGTTAC
BK569 (ORF sntB nest R)	TCGTCGTGCTCTCCTTATCATG
OZG73	ATGGTGAGCAAGGGCGAGAG
OZG74	GGTGGTGGTGCTGCAAGTGTAGCCATCGTGGCG ATGGAGCGCATGATATAG
OZG75	ATG GCC GAC AAG CAG AAG AAC
OZG76	ACGAGTTCCCACCGGGCCCATCTCAAACATGTGGTTCA GACCT CTGTTTCAG
OZG387	CGTGGCGATGGAGCGCATGATATA
OZG388	GTGGTTCATGACCTTCTGTTTCAGGTC
OZG548 (kdmB-A) for TAP	AGCAAACTCATGTTTACAACCAATG
OZG549 (kdmB-B) for TAP	CTGTGACAGGTACCATGGAAAGTG
OZG550 (kdmB-C) for TAP	CCATCTTCTCTTACCACCGCTACCACCCGCAGTGGCTTCGACTTCCG
OZG551 (kdmB-D) for TAP	CTCTACATGAGCATGCCCTGCCCCTGATTCCATCTGCCTCATTCAAC ATC
OZG552 (kdmB-E) for TAP	GAAGGACATTCTGGGATCACCG
OZG553 (kdmB-F) for TAP	GTTGGGATATGCAAGGTGTGC
OZG609 (kdmB-A) del	CAGATTGCTTCAGACTGTGTTGC
OZG610 (kdmB-B SwaI)	TTATTTAAATGGCTACTCAATGTACGTTTGC

OZG611 (kdmB-C) del	CGTTACCAATGGGATCCCGTAATCAATTGTCTTGGGTGCGATAGCTGG
OZG612 (kdmB-D) del	GACAGTATAATACAAACAAAGATGCAAGATTCCATCTGCCTCATTCAACATC
OZG613 (kdmB-E SwaI)	TTATTTAAATGAAGGACATTCTGGGATCACCG
OZG670 (kdmB for nyfp)	GCGCTCCATCGCCACGATGGTGGCTCCGGCTGCAATG
OZG671 (kdmB stop for *Swa*I)	atgcgaacccgtATTTTTAAGCGGCCGCAGTGGCTTC
OZG674 (nYFP for pmeI)	GCGCCCGCCATCGTTTATGGTGAGCAAGGGCGAGGAG
OZG677 (cYFP for pmeI)	GCGCCCGCCATCGTTTATGGCCGACAAGCAGAAGAAC
OZG694 (UP2 end of pyrG&pyroA)	CTGTCTGAGAGGAGGCACTGAT
OZG695 (UP3 head for pyrG deletion)	GCCTCAAACAATGCTCTTCA
OZG696 (UP3 head for pyroA deletion)	GGACATCAGATGCTGGATTAC
OZG752 (sntB SwaI 5)	TCGAGCTCGGTACCCATTTAAATCCCAGTCGATCACTGGGTTAC
OZG753 (sntB ptrA 5)	GATCCCGTAATCAATTCCCTCCGACGCGACAAGAAAAG
OZG754 (sntB ptrA 3)	AAACAAAGATGCAAGAGTCGAATACGTCAAACCTATTGA
OZG755 (sntB SwaI 3)	ACTCTAGAGGATCCCCATTTAAATTCGTCGTGCTCTCCTTATCATG
OZG860 (cyfp-sntB)	GAAGGTCATGAACCACATGTCCTCGGATAGGTCACC
OZG861 (nyfp-sntB)	GCGCTCCATCGCCACGATGTCCTCGGATAGGTCACC
OZG862 (sntB-niit stop)	gtatcctcgtcagtTTTCACCACAGCCTGGTGTCGAG
OZG863 (cyfp-ecoA)	GAAGGTCATGAACCACATGCCGTGGACTGTAACAAATTC
OZG864 (nyfp-ecoA)	GCGCTCCATCGCCACGATGCCGTGGACTGTAACAAATTC
OZG865 (ecoA-niit stop)	gtatcctcgtcagtTTTCAACTCTCCTTGTAGACATGC
OZG916 (GGGSGG amplifier for all cassettes)	GGAGGTGGTAGCGGTGGT
OZG917 (natR 3´ prime)	TCAGGGGCAGGGCATGCTCA

OZG1033 (rpdA 5 UTR PmeI)	TTCGAGCTCGGTACCCGTTTAAACGCACAGGAAAGGAACACGAAG
OZG1034 (rpdA GFP & TAP fuser)	TACCACCGCTACCACCCTCCTTTGCGGCTTCGGGTTG
OZG1035 (rpdA natR)	CATGCCCTGCCCCTGAGGACTAGCGCAGCAATTTTGG
OZG1036 (rpdA 3 UTR PmeI)	ACTCTAGAGGATCCCCGTTTAAACCTCTCTCCATGTATTGACGCAAG
OZG1037 (sntB 5 UTR PmeI)	TTCGAGCTCGGTACCCGTTTAAACGCCTACCTTGTCTGTCTACTG
OZG1039 (sntB GFP & TAP fuser)	TACCACCGCTACCACCGGACAGGAGGTTCTTCAGCG
OZG1040 (sntB natR)	CATGCCCTGCCCCTGACTTGGTCGTAGAGAGATCAAGG
OZG1041 (sntB 3 UTR PmeI)	ACTCTAGAGGATCCCCGTTTAAACCTAGCTGGATTCTGGAAGCAG
OZG1042 (ecoA 5 UTR PmeI)	TTCGAGCTCGGTACCCGTTTAAACGGAGCGAGCGCGATGGTTG
OZG1043 (ecoA GFP & TAP fuser)	TACCACCGCTACCACCACTCTCCTTGTAGACATGCCA
OZG1044 (ecoA natR)	CATGCCCTGCCCCTGATGGTTTGTGCACAATTGTCATTCG
OZG1045 (ecoA 3 UTR PmeI)	ACTCTAGAGGATCCCCGTTTAAACGCATTCCTGAGAAGCCTGTTG
OZG1077 (pyroA 5 for TET plasmid)	cctcggcagatctgcaGGACATCAGATGCTGGATTAC
OZG1184 (pyroA 3 for TET plasmid)	aacaaaagctgggtacATTTAAATCTGTCTGAGAGGAGGCACTGAT
OZG1237 (EcoA S41A/S45A RV)	TTTCAGCCGCGTCCGCCTCGCAATCCGCCAAAACGCGTCGTTTCTTG GC
OZG1238 (EcoA S41A/S45A FWD)	CGGACGCGGCTGAAAAGAGCC
OZG1239 (EcoA S41D RV)	GTCCGACTCGCAATCGTCCAAAACGCGTCGTTTCTTGGC
OZG1240 (EcoA S41D FWD)	GATTGCGAGTCGGACGCGG
OZG1241 (EcoA S45D RV)	CTCGCAATCCGACAAAACGC
OZG1242 (EcoA S45D FWD)	TTGTCGGATTGCGAGGATGACGCGGCTGAAAAGAGCC
OZG1243 (EcoA S41D/S45D RV)	CTTTTCAGCCGCGTCATCCTCGCAATCGTCCAAAACGCGTCGTTTCT TGG
OZG1244 (EcoA S41D/S45D FWD)	GACGCGGCTGAAAAGAGCC
OZG1251 (pyroAFWDSem1)	TCTCAGATTTGAATTCGGACATCAGATGCTGGATTAC

OZG1252 (pyroARVSem1)	CACAGCCATTTACATACTGTCTGAGAGGAGGCACTGAT
OZG1256 (EcoA S45A RV)	TCTTTTCAGCCGCGTCCGCCTCGCAATCCGACAAAACGCG
OZG1257 (EcoA S45A FWD)	GACGCGGCTGAAAAGAGCCTTGAG
OZG1258 (ecoA S41A RV)	CGACTCGCAATCCGCCAAAACGCGTCGTTTCTTGGCC
OZG1259 (ecoA S41A FWD)	GCGGATTGCGAGTCGGACGCGG
gpdA North. FWD	TTGAGACCTACGACGAGGGT
gpdA North. RV	TGACGGCATCCTTGATCTGG
ecoA North. FWD	CCACAGAGGTCTCATGATGA
ecoA North. RV	CCTTCATGTGCAAGAACACCTTG
veA North. FWD	CGAAATGTATGGTGGCAGC
veA North. RV	GCTGTAGACGATAAAGGGG
laeA North. FWD	TCAAGGCAGAACTCGGATG
laeA North. RV	GACAATCGTACCAATGCCG
stcU North. FWD	ACCGTCTCGATGGAAAAG
stcU North. RV	GCGTTGACCGTGATCTTC
stcN North. FWD	GGTAGACAAGGCCCTCGA
stcN North. RV	CCCATCAACTGGCTGTGG

Supplemental Table S2. Plasmids employed in this study.

**Plasmid**	**Description**	**Reference**
pUC19	*E. coli* cloning plasmid with *bla* (ampicillin resistance gene) gene	Thermo Fisher
pJET1.2	*E. coli* cloning plasmid with *bla* (ampicillin resistance gene) gene	Thermo Fisher
pME4567	*sem1Δ::ptrA* cassette in pJET1.2 plasmid	(1)
pOSB114	*Pme*I*::AfpyroA::Swa*I inserted in *Sma*I site of pUC19 (complementation plasmid)	(2)
pCH008	*tetO7::Pmin::ptrA* cassette containing plasmid	(3)
pBK1	*rpdA* (AN4493) *3xha::AfpyrG* cassette with *Pme*I in *Sma*I site of pUC19	This study
pBK2	*sntB* (AN9507) *3xha::AfpyrG* cassette with *Pme*I in *Sma*I site of pUC19	This study
pBK3	*ecoA* (AN10336) *3xha::AfpyrG* cassette with *Pme*I in *Sma*I site of pUC19	This study
pBK11	*kdmB* (AN8211) *3xha::AfpyrG* cassette with *Pme*I in *Sma*I site of pUC19	This study
pBK32	*tetO7::Pmin::ecoA/pyroA* cassette in *Sma*I site of pUC19	This study
pBK33	*tetO7::Pmin::rpdA/pyroA* cassette in *Sma*I site of pUC19	This study
pBK53	*kdmBΔ::AfpyrG* cassette in *Sma*I site of pUC19	This study
pBK74	*kdmB* genomic locus in *Swa*I site of pOSB114	This study
pBK75	*sntB* genomic locus in *Swa*I site of pOSB114	This study
pBK87	*sntB::3xha::AfpyrG* cassette with *Pme*I in *Sma*I site of pUC19	This study
pBK128	*sntBΔ::pyroA* cassette in *Sma*I site of pUC19	This study
pOB226	*kdmBΔ::ptrA* cassette with in pJET1.2	This study
pOB254	*sntBΔ::ptrA* cassette in pJET1.2	This study
pOB282	*N-yfp::kdmB* genomic locus in *Swa*I site of pSK353	This study
pOB283	*C-yfp::kdmB* genomic locus in *Swa*I site of pSK353	This study
pOB302	CYFP OZG677/OZG388 + OZG857/OZG859 *rpdA* in *Pme*I site of pOB282A	This study
pOB303	NYFP OZG674/OZG387 + OZG858/OZG859 *rpdA* in *Pme*I site of pOB283A	This study

pOB304	CYFP OZG677/OZG388 + OZG860/OZG862 *sntB* in *Pme*I site of pOB282A	This study
pOB305	NYFP OZG674/OZG387 + OZG861/OZG862 *sntB* in *Pme*I site of pOB283A	This study
pOB306	CYFP OZG677/OZG388 + OZG863/OZG865 *ecoA* in *Pme*I site of pOB282A	This study
pOB307	NYFP OZG674/OZG387 + OZG864/OZG865 *ecoA* in *Pme*I site of pOB283A	This study
pOB430	3X HA with GGGSGG linker *trpC pyrG* in *Sma*I of pUC19	This study
pOB485	*rpdA sgfp::natR* cassette with *Pme*I in *Sma*I site of pUC19	This study
pOB486	*rpdA ctap::natR* cassette with *Pme*I in *Sma*I site of pUC19	This study
pOB487	*sntB sgfp::natR* cassette with *Pme*I in *Sma*I site of pUC19	This study
pOB488	*sntB ctap::natR* cassette with *Pme*I in *Sma*I site of pUC19	This study
pOB489	*ecoA sgfp::natR* cassette with *Pme*I in *Sma*I site of pUC19	This study
pOB490	*ecoA ctap::natR* cassette with *Pme*I in *Sma*I site of pUC19	This study
pOB508	*tetO7::Pmin::pyroA* cassette in *Sma*I site of pUC19 for ectopic expression	This study
pOB549	*tetO7::Pmin::pyroA* cassette in *Sma*I site of pUC19 for promoter replacement	This study
pOB559	*ecoA* (AN10336) *3xha::AfpyrG* cassette with *Pme*I in *Sma*I site of pUC19 (41Ser>41Ala)	This study
pOB560	*ecoA* (AN10336) *3xha::AfpyrG* cassette with *Pme*I in *Sma*I site of pUC19 (45Ser>45Ala)	This study
pOB561	*ecoA* (AN10336) *3xha::AfpyrG* cassette with *Pme*I in *Sma*I site of pUC19 (41and 45 Ser>41and 45 Ala)	This study
pOB562	*ecoA* (AN10336) *3xha::AfpyrG* cassette with *Pme*I in *Sma*I site of pUC19 (41Ser>41Asp)	This study
pOB563	*ecoA* (AN10336) *3xha::AfpyrG* cassette with *Pme*I in *Sma*I site of pUC19 (45Ser>45 Asp)	This study
pOB564	*ecoA* (AN10336) *3xha::AfpyrG* cassette with *Pme*I in *Sma*I site of pUC19 (41and 45 Ser>41and 45 Asp)	This study
pOB566	*sem1Δ::pyroA* cassette in pJET1.2 plasmid	This study

Supplemental Table S3. Strains employed in this study.

**Strain**	**Genotype**	**Reference**
AGB551	*nkuAΔ::argB,pyrG89,pyroA4,veA+*	(4)
ANBK1	*rpdA::3xHA::AfpyrG;nkuAΔ::argB, pyroA4,veA+*	This study
ANBK2	*sntB::3xHA::AfpyrG;nkuAΔ::argB, pyroA4,veA+*	This study
ANBK3	*ecoA::3xHA::AfpyrG;nkuAΔ::argB, pyroA4,veA+*	This study
ANBK5	*kdmB::ctap::natR;nkuAΔ::argB, pyrG89, pyroA4,veA+*	This study
ANBK11	*kdmB::3xHA::AfpyrG;nkuAΔ::argB, pyroA4,veA+*	This study
ANBK32	*tetO7::Pmin::ecoA/pyroA; nkuAΔ::argB,pyrG89,veA+*	This study
ANBK33	*tetO7::Pmin::rpdA/pyroA; nkuAΔ::argB,pyrG89,veA+*	This study
ANBK54	*ecoA::ctap::natR, kdmBΔ::ptrA, nkuAΔ::argB, pyrG89, pyroA4,veA+*	This study
ANBK55	*rpdA::ctap::natR, kdmBΔ::ptrA, nkuAΔ::argB, pyrG89, pyroA4,veA+*	This study
ANBK56	*sntB::ctap::natR, kdmBΔ::ptrA, nkuAΔ::argB, pyrG89, pyroA4,veA+*	This study
ANBK67	*kdmB::ctap::natR, sntBΔ::ptrA, nkuAΔ::argB, pyrG89, pyroA4,veA+*	This study
ANBK68	*ecoA::ctap::natR, sntBΔ::ptrA, nkuAΔ::argB, pyrG89, pyroA4,veA+*	This study
ANBK69	*rpdA::ctap::natR, sntBΔ::ptrA, nkuAΔ::argB, pyrG89, pyroA4,veA+*	This study
ANBK80	*kdmBΔ::ptrA, rpdA::sgfp::natR, h2A::mRFP::pyroA, nkuAΔ::argB, bioA, veA+*	This study
ANBK81	*kdmBΔ::ptrA, sntB::sgfp::natR, h2A::mRFP::pyroA, nkuAΔ::argB, bioA, veA+*	This study
ANBK82	*kdmBΔ::ptrA, ecoA::sgfp::natR, h2A::mRFP::pyroA, nkuAΔ::argB, bioA, veA+*	This study
ANBK83.1	*kdmBΔ::ptrA, ecoA::3xHA::AfpyrG, nkuAΔ::argB, pyroA4,veA+*	This study
ANBK84.2	*kdmBΔ::ptrA, rpdA::3xHA::AfpyrG, nkuAΔ::argB, pyroA4,veA+*	This study
ANBK85.4	*kdmBΔ::ptrA, sntB::3xHA::AfpyrG, nkuAΔ::argB, pyroA4,veA+*	This study
ANBK98	*sntBΔ::ptrA, kdmB::sgfp::natR, h2A::mRFP::AfpyrG, pyroA4 nkuAΔ::argB, veA+*	This study
ANBK99	*sntBΔ::ptrA, ecoA::sgfp::natR,* ^{P}*biA::h2A::mRFP::pyroA::*^{t}*biA, pyrG89, nkuAΔ::argB, veA+*	This study

ANBK100	*sntBΔ::ptrA, rpdA::sgfp::natR, ^{P}biA::h2A::mRFP::AfpyrG::^{T}biA, pyrG89, nkuAΔ::argB, veA+*	This study
ANBK101.1	*ecoA::3xHA::AfpyrG, sntBΔ::ptrA, nkuAΔ::argB, pyroA4,veA+*	This study
ANBK102.1	*rpdA::3xHA::AfpyrG, sntBΔ::ptrA, nkuAΔ::argB, pyroA4,veA+*	This study
ANBK103.1	*kdmB::3xHA::AfpyrG, sntBΔ::ptrA, nkuAΔ::argB, pyroA4,veA+*	This study
ANBK112	*sntBΔ::pyroA, kdmBΔ::ptrA, ecoA::3xHA::AfpyrG, nkuAΔ::argB ,veA+*	This study
ANBK113	*kdmB::sgfp::natR;nkuAΔ::argB, pyrG89, pyroA4,veA+*	This study
ANOB302	*^{p}niiA::nyfp::kdmB::niiA^{T}- ^{p}niaD::cyfp::rpdA::niaD^{T}, A.f. pyrG; ^{p}gpdA::mrfp::h2A, ^{p}gpdA::natR; pyroA4, pyrG89*	This study
ANOB303	*^{p}niiA::nyfp::rpdA::niiA^{T}- ^{p}niaD::cyfp::kdmB::niaD^{T}, A.f. pyrG; ^{p}gpdA::mrfp::h2A, ^{p}gpdA::natR; pyroA4, pyrG89*	This study
ANOB304	*^{p}niiA::nyfp::kdmB::niiA^{T}- ^{p}niaD::cyfp::sntB::niaD^{T}, A.f. pyrG; ^{p}gpdA::mrfp::h2A, ^{p}gpdA::natR; pyroA4, pyrG89*	This study
ANOB305	*^{p}niiA::nyfp::sntB::niiA^{T}- ^{p}niaD::cyfp::kdmB::niaD^{T}, A.f. pyrG; ^{p}gpdA::mrfp::h2A, ^{p}gpdA::natR; pyroA4, pyrG89*	This study
ANOB306	*^{p}niiA::nyfp::kdmB::niiA^{T}- ^{p}niaD::cyfp::ecoA::niaD^{T}, A.f. pyrG; ^{p}gpdA::mrfp::h2A, ^{p}gpdA::natR; pyroA4, pyrG89*	This study
ANOB307	*^{p}niiA::nyfp::ecoA::niiA^{T}- ^{p}niaD::cyfp::kdmB::niaD^{T}, A.f. pyrG; ^{p}gpdA::mrfp::h2A, ^{p}gpdA::natR; pyroA4, pyrG89*	This study
ANOB485	*rpdA::sgfp::natR;nkuAΔ::argB, pyrG89, pyroA4,veA+*	This study
ANOB486	*rpdA::ctap::natR;nkuAΔ::argB, pyrG89, pyroA4,veA+*	This study
ANOB487	*sntB::sgfp::natR;nkuAΔ::argB, pyrG89, pyroA4,veA+*	This study
ANOB488	*sntB::ctap::natR;nkuAΔ::argB, pyrG89, pyroA4,veA+*	This study
ANOB489	*ecoA::sgfp::natR;nkuAΔ::argB, pyrG89, pyroA4,veA+*	This study
ANOB490	*ecoA::ctap::natR;nkuAΔ::argB, pyrG89, pyroA4,veA+*	This study
ANOB559	*ecoA^{S41A}::3xHA::AfpyrG;nkuAΔ::argB, pyroA4,veA+*	This study
ANOB560	*ecoA^{S45A}::3xHA::AfpyrG;nkuAΔ::argB, pyroA4,veA+*	This study
ANOB561	*ecoA$^{S41A/S45A}$::3xHA::AfpyrG;nkuAΔ::argB, pyroA4,veA+*	This study
ANOB562	*ecoA^{S41D}::3xHA::AfpyrG;nkuAΔ::argB, pyroA4,veA+*	This study
ANOB563	*ecoA^{S45D}::3xHA::AfpyrG;nkuAΔ::argB, pyroA4,veA+*	This study
ANOB564	*ecoA$^{S41D/S45D}$::3xHA::AfpyrG;nkuAΔ::argB, pyroA4,veA+*	This study
ANOBKD559	*ecoA^{S41A}::3xHA::AfpyrG;kdmBΔ::ptrA;nkuAΔ::argB, pyroA4,veA+*	This study
ANOBKD560	*ecoA^{S45A}::3xHA::AfpyrG;kdmBΔ::ptrA;nkuAΔ::argB, pyroA4,veA+*	This study

ANOBKD561	*ecoA*$^{S41A/S45A}$*::3xHA::AfpyrG;kdmBΔ::ptrA;nkuAΔ::argB, pyroA4,veA+*	This study
ANOBKD562	*ecoA*^{S41D}*::3xHA::AfpyrG;kdmBΔ::ptrA;nkuAΔ::argB, pyroA4,veA+*	This study
ANOBKD563	*ecoA*^{S45D}*::3xHA::AfpyrG;kdmBΔ::ptrA;nkuAΔ::argB, pyroA4,veA+*	This study
ANOBKD564	*ecoA*$^{S41D/S45D}$*::3xHA::AfpyrG;kdmBΔ::ptrA;nkuAΔ::argB, pyroA4,veA+*	This study
ANBK83.1.566	*Sem1Δ::pyroA;kdmBΔ::ptrA,ecoA::3xHA::AfpyrG, nkuAΔ::argB, pyroA4,veA+*	This study

Supplemental Table S4. A summary of the MEME-ChIP analysis on the common binding sites of the KERS subunits KdmB, EcoA, RpdA and SntB.

Motif Enriched in KERS binding sites	E-value	Known or Similar Motifs
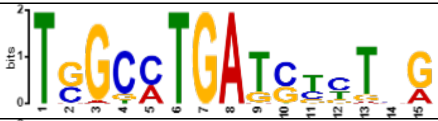	4.8e-029	-
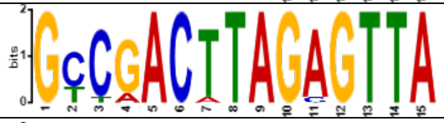	2.6e-016	TBF1 (MA0403.1)
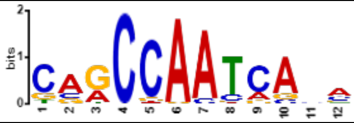	8.6e-015	HAP3 (MA0314.1)<br>HAP5 (MA0316.1)<br>HAP2 (MA0313.1)
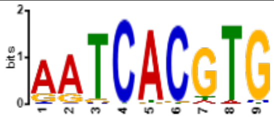	5.7e-011	TYE7 (MA0409.1)<br>CBF1 (MA0281.1)<br>RTG3 (MA0376.1)
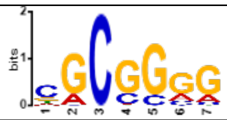	3.8e-008	SUT1 (MA0399.1)
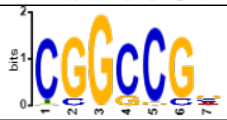	2.2e-006	RDS1 (MA0361.1)
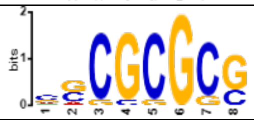	5.1e-006	RSC30 (MA0375.1)
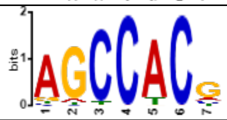	2.0e-005	MET32 (MA0334.1)<br>CRZ1 (MA0285.1)
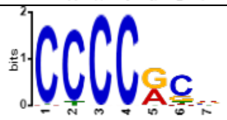	2.5e-005	MIG3 (MA0339.1)
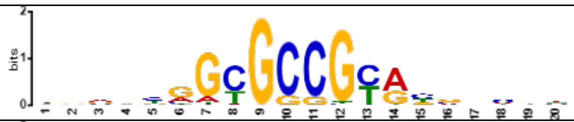	2.7e-005	STP2 (MA0395.1)
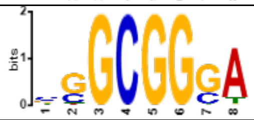	4.5e-005	UGA3 (MA0410.1)
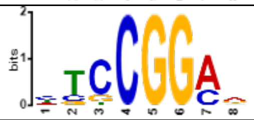	5.7e-005	YER184C (MA0424.1)
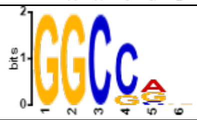	4.2e-004	SKN7 (MA0381.1)
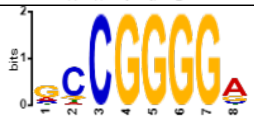	5.7e-004	NHP10 (MA0344.1)

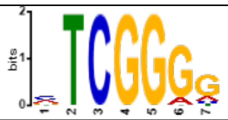	1.1e-003	RDS2 (MA0362.1)
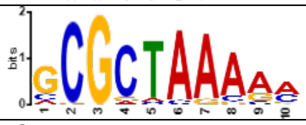	1.7e-003	-
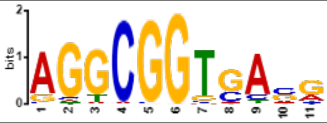	3.3e-003	CHA4 (MA0283.1)<br>DOT6 (MA0351.1)<br>TOD6 (MA0350.1)
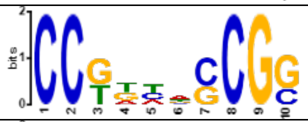	7.4e-003	LEU3 (MA0324.1)
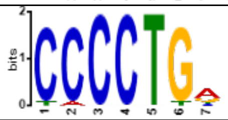	8.4e-003	REI1 (MA0364.1)
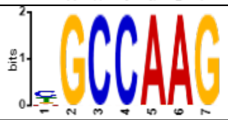	4.5e-002	RIM101 (MA0368.1)

Supplemental Data

Supplemental Data S1 LC-MS/MS protein lists combined from two biological replicates of KdmB^{TAP} purification. Non-specific peptide contaminants were filtered out by using WT as a negative control.

Supplemental Data S2 LC-MS/MS protein lists combined from two biological replicates of EcoA^{TAP} purification. Non-specific peptide contaminants were filtered out by using WT as a negative control.

Supplemental Data S3 LC-MS/MS protein lists combined from two biological replicates of RpdA^{TAP} purification. Non-specific peptide contaminants were filtered out by using WT as a negative control.

Supplemental Data S4 LC-MS/MS protein lists combined from two biological replicates of SntB^{TAP} purification. Non-specific peptide contaminants were filtered out by using WT as a negative control.

Supplemental Data S5 LC-MS/MS protein lists combined from two biological replicates of KdmB^{GFP} purification. Non-specific peptide contaminants were filtered out by using GFP expressing WT as a negative control.

Supplemental Data S6 LC-MS/MS protein lists combined from two biological replicates of EcoA^{GFP} purification. Non-specific peptide contaminants were filtered out by using GFP expressing WT as a negative control.

Supplemental Data S7 LC-MS/MS protein lists combined from two biological replicates of RpdA^{GFP} purification. Non-specific peptide contaminants were filtered out by using GFP expressing WT as a negative control.

Supplemental Data S8 LC-MS/MS protein lists combined from two biological replicates of SntB^{GFP} purification. Non-specific peptide contaminants were filtered out by using GFP expressing WT as a negative control.

Supplemental Data S9 LC-MS/MS protein lists combined from two biological replicates of KdmB^{HA} purification. Non-specific peptide contaminants were filtered out by using WT as a negative control.

Supplemental Data S10 LC-MS/MS protein lists combined from two biological replicates of EcoA^{HA} purification. Non-specific peptide contaminants were filtered out by using WT as a negative control.

Supplemental Data S11 LC-MS/MS protein lists combined from two biological replicates of RpdA^{HA} purification. Non-specific peptide contaminants were filtered out by using WT as a negative control.

Supplemental Data S12 LC-MS/MS protein lists combined from two biological replicates of SntB^{HA} purification. Non-specific peptide contaminants were filtered out by using WT as a negative control.

Supplemental Data S13 LC-MS/MS protein lists combined from two biological replicates of $EcoA^{TAP}$, *kdmB*Δ purification. Non-specific peptide contaminants were filtered out by using WT as a negative control.

Supplemental Data S14 LC-MS/MS protein lists combined from two biological replicates of $RpdA^{TAP}$, *kdmB*Δ purification. Non-specific peptide contaminants were filtered out by using WT as a negative control.

Supplemental Data S15 LC-MS/MS protein lists combined from two biological replicates of $SntB^{TAP}$, *kdmB*Δ purification. Non-specific peptide contaminants were filtered out by using WT as a negative control.

Supplemental Data S16 LC-MS/MS protein lists combined from two biological replicates of $KdmB^{TAP}$, *sntB*Δ purification. Non-specific peptide contaminants were filtered out by using WT as a negative control.

Supplemental Data S17 LC-MS/MS protein lists combined from two biological replicates of $EcoA^{TAP}$, *sntB*Δ purification. Non-specific peptide contaminants were filtered out by using WT as a negative control.

Supplemental Data S18 LC-MS/MS protein lists combined from two biological replicates of $RpdA^{TAP}$, *sntB*Δ purification. Non-specific peptide contaminants were filtered out by using WT as a negative control.

Supplemental Data S19 MACS analysis results for KdmB, EcoA, RpdA and SntB ChIPseq data.

Supplemental Data S20 Genes and their description for each of the fifteen groups with bindings by different number of KERS proteins.

Supplemental Data S21 ChIPseq signals of RNA Pol II and KdmB used in Figure 3H.

Supplemental Data S22 Differentially expressed genes and their fold changes when comparing between primary (at 20 hr) to secondary (at 48 hr) growth stages in wildtype and the *kdmB*Δ mutant.

Supplemental Data S23 GO analysis output for different classes of genes from comparing transcriptional response of wildtype and *kdmB*Δ strains to transition from primary (20 hr) to secondary (48 hr) growth phase.

References

1. Kolog Gulko, M., Heinrich, G., Gross, C., Popova, B., Valerius, O., Neumann, P., Ficner, R. and Braus, G.H. (2018) Sem1 links proteasome stability and specificity to multicellular development. *PLoS Genet*, **14**, e1007141.
2. Elramli, N., Karahoda, B., Sarikaya-Bayram, O., Frawley, D., Ulas, M., Oakley, C.E., Oakley, B.R., Seiler, S. and Bayram, O. (2019) Assembly of a heptameric STRIPAK complex is required for coordination of light-dependent multicellular fungal development with secondary metabolism in Aspergillus nidulans. *PLoS Genet*, **15**, e1008053.
3. Helmschrott, C., Sasse, A., Samantaray, S., Krappmann, S. and Wagener, J. (2013) Upgrading fungal gene expression on demand: improved systems for doxycycline-dependent silencing in Aspergillus fumigatus. *Appl Environ Microbiol*, **79**, 1751-1754.
4. Bayram, O., Bayram, O.S., Ahmed, Y.L., Maruyama, J., Valerius, O., Rizzoli, S.O., Ficner, R., Irniger, S. and Braus, G.H. (2012) The *Aspergillus nidulans* MAPK module AnSte11-Ste50-Ste7-Fus3 controls development and secondary metabolism. *PLoS Genet*, **8**, e1002816.
